# Non-invasive bioinert room-temperature quantum sensor from silicon carbide qubits

**DOI:** 10.1038/s41563-025-02382-9

**Published:** 2025-11-06

**Authors:** Pei Li, Ji-Yang Zhou, Song Li, Péter Udvarhelyi, Jin-Shi Xu, Chuan-Feng Li, Bing Huang, Guang-Can Guo, Adam Gali

**Affiliations:** 1 School of Integrated Circuit Science and Engineering, Tianjin University of Technology, Tianjin, China; 2 Beijing Computational Science Research Center, Beijing, China; 3 HUN-REN Wigner Research Centre for Physics, Budapest, Hungary; 4 Laboratory of Quantum Information, University of Science and Technology of China, Hefei, China; 5 Anhui Province Key Laboratory of Quantum Network, University of Science and Technology of China, Hefei, China; 6 CAS Center For Excellence in Quantum Information and Quantum Physics, University of Science and Technology of China, Hefei, China; 7 Department of Atomic Physics, Institute of Physics, Budapest University of Technology and Economics, Budapest, Hungary; 8 Department of Chemistry and Biochemistry, University of California Los Angeles, Los Angeles, CA USA; 9 Hefei National Laboratory, University of Science and Technology of China, Hefei, China; 10 Department of Physics, Beijing Normal University, Beijing, China; 11 MTA-WFK Lendület ‘Momentum’ Semiconductor Nanostructures Research Group, Budapest, Hungary

**Keywords:** Quantum metrology, Electronic properties and materials, Surfaces, interfaces and thin films

## Abstract

Room-temperature shallow defect spin qubits acting as a quantum sensor with favourable properties towards the biological environment are sought after, with promising impacts on bioimaging, radical detection and nanoscale nuclear spin sensing. Here we show that alkene-terminated silicon carbide hosting divacancy qubits located a few nanometres below the surface leads to a stable operation with superior sensitivity in which the host is a bioinert semiconductor with existing wafer-scale chip technology. The read-out of the qubit occurs at near-infrared wavelengths, which exhibit a minimum absorption by the organic molecules or water. We show that the divacancy qubit can realize multiple quantum sensor schemes under ambient conditions in which the suggested surface termination can be readily tailored towards the desired application. The combination of the paramount host, surface functionalization and qubit properties may significantly advance room-temperature quantum sensing, as well as provide a platform for quantum simulation and optoelectronic devices.

## Abstract (web summary)

Alkene-terminated silicon carbide surfaces are proposed as a room-temperature divacancy spin qubit quantum sensor suitable for bioimaging and nanoscale nuclear spin sensing.

## Main

Room-temperature operation of defect qubits contributes to sustainable green technology by avoiding energy consumption for cooling and it is a quest for quantum sensor applications in the field of in vivo biological and medical applications. Room-temperature operation poses stringent criteria both for defect types and the host materials because the spin–phonon couplings with the effective (quasi)local phonons should lie at high frequencies^1^, which restricts materials and defects with light elements in the second row (for example, diamond from carbon and boron nitride from boron and nitrogen) or the combination of the second and third rows (for example, silicon carbide (SiC) from carbon and silicon) in the periodic table. Experimentally confirmed room-temperature single-defect spins with high optically detected magnetic resonance (ODMR) contrast are the negatively charged nitrogen-vacancy (NV) centre in diamond^2–4^ and the divacancy in SiC^5–7^ that produce deep levels in these wide-bandgap materials. Indeed, a leading contender in quantum (bio)sensor applications is the diamond NV centre^8^. However, the diamond NV centre can only be efficiently excited by green light (typically at 532 nm) where the absorption of organic molecules and water is very efficient, leading to autofluorescence and heating^9^. This intrinsic property of the diamond NV centre cannot be circumvented; therefore, there is an urgent quest to seek an alternative solution.

The neutral divacancy and divacancy-related defect qubits in SiC^5,6,10^ do not require extrinsic defects for doping; their excitation and emission wavelengths fall in the so-called second biological window in the near-infrared regime in which autofluorescence from the organic species and water is minimal, making them highly suitable for biocompatible quantum sensing applications. Compared with silicon vacancies, which have also been demonstrated as quantum sensors^11–15^, divacancies offer superior stability^16^, similar coherence times (${T}_{2}^{* }\approx 30-50$ µs in isotope-engineered SiC)^17,18^ but substantially higher ODMR contrasts (10%–20% versus 0.5%), saturating photon counts (~150 versus ~10 kcps)^7,19^ and robustness in harsh environments^20^. These properties, coupled with their neutrality, make divacancies particularly advantageous for quantum sensing. Divacancies, aggregates of adjacent carbon and silicon vacancies, are typically created by implantation and annealing; thus, divacancy qubits can be readily engineered near the surface of SiC. However, only a few studies reported shallow defect qubits in SiC^18^. In these studies, SiC is thermally oxidized, which leads to the formation of an insulating amorphous SiO_2_ (a-SiO_2_) layer—the typical insulator in SiC-based electronics devices^21^. Unlike silicon, where a stable thermal oxide can be readily grown, forming a high-quality oxide layer on SiC is challenging. Standard thermal oxidation processes often result in many interface defects that can introduce defect levels within the bandgap of SiC and act as traps for carriers, even after post-treatments^22–25^. For instance, single-photon emitters on 532-nm illumination were found at the interface with unknown origin^26,27^, where the number of emitters can be reduced but not fully eliminated by post-oxidation treatments^28,29^. We note that simple hydrogen-terminated SiC starts to oxidize at room temperature in air within half an hour^30,31^ and an ~1-nm-thick oxygen-rich layer is developed after 2 days (ref. ^31^). Controlling the oxidation of SiC or finding an alternative robust solution for surface termination is the most urgent and unresolved issue to advance bioquantum sensors.

In this study, we systematically investigate the colour centres at the interface of SiC and a-SiO_2_ that occur due to the incomplete oxidation of SiC. From first-principles simulations, we find that these colour centres primarily originate from carbon clusters with various compositions that introduce deep levels in the bandgap and yield emission from the near-infrared region to the blue region in the visible region. We conclude that the stochastic nature of the formation of these colour centres and their inhomogeneous environment renders them unsuitable for quantum applications. Instead, they interfere with the operation of the engineered divacancy spin quantum sensor; thus, a radically different surface treatment is necessary to secure a stable operation. We find that the alkene-terminated SiC surface produces an ideal environment for the divacancy spin quantum sensor, which can be produced by low-temperature chemistry and it is stable under ambient conditions^32^. We demonstrate that spin-relaxometry quantum sensing protocols can be carried out with substantial contrast in *T*_1_ times in the presence of paramagnetic species. The well-established magneto-optical and spin parameters in isotopically engineered materials^17^ imply that the sensitivity reaches $\sim 13\,\,\text{nT}/\sqrt{\text{Hz}\,}$ in quantum nuclear magnetic resonance (qNMR) experiments. All of these quantum optics protocols can be carried out with such optical excitation and emission wavelengths that minimally perturb organic systems, and the surface can be straightforwardly functionalized for target bioapplications with currently available chemistry methods. We demonstrate these principles on single-divacancy-related qubits engineered ~1–2 nm beneath a non-optimized SiC surface, achieving a sensitivity of approximately $56\,\,\text{nT}/\sqrt{\text{Hz}\,}$.

## Results and discussion

### 4H-SiC–a-SiO_2_ interface

**Fig. 1:**
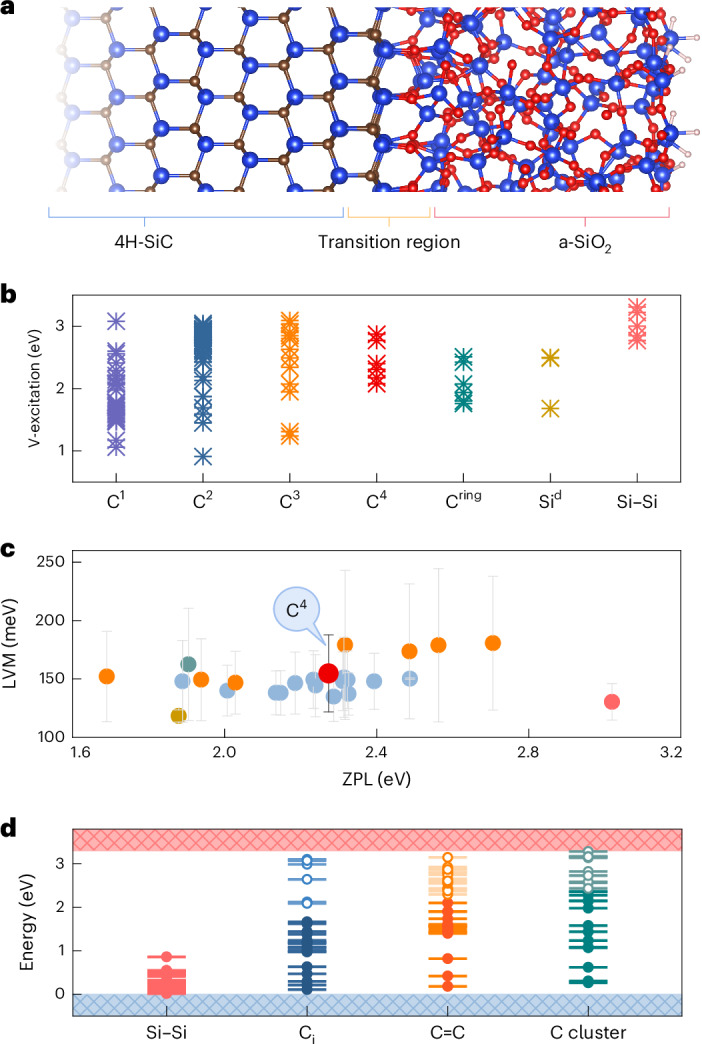
Atomic structure and interface-defect-related properties of the 4H-SiC–a-SiO_2_ interface. **a**, Model of the 4H-SiC–a-SiO_2_ interface. **b**, Vertical excitation (V-excitation) energies of C-related defects involving up to four carbon atoms (C^*n*^, where *n* is the number of C atoms), Si dangling bond (Si^d^) and Si–Si dimer defects. **c**, Range of LVM (vertical bars) and ZPL (symbols) values for the most stable defects in **b**. The symbols mark the ZPL energies and are placed at the midpoint of each LVM range. Vertical bars indicate the corresponding LVM energy ranges (min–max). **d**, Calculated Kohn–Sham defect levels of Si–Si dimer defects, carbon interstitials (C_i_), C-related defects (C=C) with *sp*^2^ hybridization and C-cluster defects. Source data

SiC is a wide-bandgap material that may exist in various crystalline structures called polytypes. The technologically most advanced polytype is the so-called 4H polytype (4H-SiC), which has an ~3.3-eV bandgap at room temperature. The atomistic model of the 4H-SiC–a-SiO_2_ interface is shown in Fig. 1a. During oxidation of the SiC surface, the reaction between oxygen and silicon can lead to the formation of a SiO_2_ unit, which is a key component of the oxide layer. As oxygen atoms react with silicon atoms, carbon atoms become exposed. The exposed carbon atoms can nucleate into carbon clusters or graphene-like structures on the SiC surface. These clusters may grow by incorporating additional carbon atoms from nearby vacancies or the surrounding environment. Apart from carbon clusters, the interface transition region also encompasses paramagnetic dangling bonds, Si–Si dimers and oxygen-related defects. For the C-related defects (C^*n*^, where *n* is the number of C atoms), the configurations exhibit increased complexity due to structural changes in the interface transition and diverse combinations of defect atoms. These distinct localized structures of defects lead to significant variations in the distribution of defect levels among them (Fig. 1). For carbon-related defects, the zero-phonon line (ZPL) is predominantly distributed in the range of 1.7–2.7 eV. As for Si–Si dimer defects, the ZPL peak falls in the deep blue region. These observations align with previous experimental findings on photoluminescence (PL) centres at the interface with ZPL peaks ranging from 1.5 to 2.5 eV on illumination with 532-nm and 633-nm lasers, respectively^33,34^. The concentration of these PL centres may be reduced to an isolated level, resulting in quantum emission at the 4H-SiC–a-SiO_2_ interface^27^ that show sharp high-energy local vibration modes (LVMs) between 120 and 200 meV. We find that many of the considered carbon-related emitters (around 100 configurations; Supplementary Note 1) exhibit high-energy LVMs. In particular, the carbon cluster consisting of four carbon atoms produces a very similar PL spectrum to the observed one, with a ZPL peak at around 2.1 eV (ref. ^27^). We note that the PL spectra substantially vary with the actual environment. Because these carbon clusters are formed during the thermal oxidation of SiC (which is a stochastic process with different environments), it is principally impossible to form indistinguishable colour centres; thus, it might be difficult to use them as a resource for quantum technologies. We note that most of these colour centres occur in their neutral charge state with a bound exciton excited state^35^ and they have a singlet ground state.

### Carbon-chain interface

**Fig. 2:**
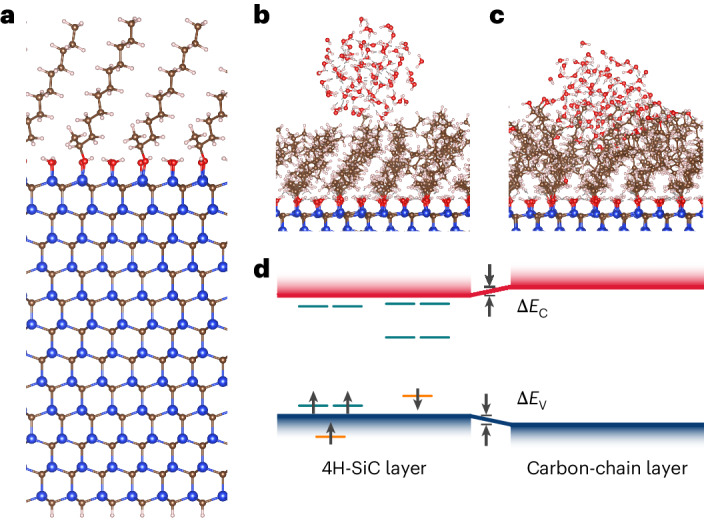
Structural properties of alkene-modified 4H-SiC interface. **a**, Model of the alkene-modified interface. **b**,**c**, Hydrophobicity and hydrophilicity of the carbon-chain group with a methyl group (**b**) and an alcohol group (**c**) at the end of the carbon chain. **d**, Band offset (Δ*E*_V_ and Δ*E*_C_) between SiC and carbon-chain layers. Calculated Kohn–Sham defect levels of divacancy (V_Si_ − V_C_) in SiC are also shown. Source data

To alleviate the detrimental influence of interface defects—with myriad deep levels (Fig. 1d)—on the properties of defect qubits in SiC, we suggest a passivation technique using 1-alkene, specifically CH_2_=CHC_11_H_23_, on the SiC surface. This approach has been successfully demonstrated^32^, which includes the selective etching of oxide in a hydrogen fluoride solution and alkene reaction at about 130 °C, which is a much lower temperature than the activation temperature for the diffusion of known defect qubits in SiC. The atomistic picture of an alkene-modified interface is illustrated in Fig. 2a. The length of the carbon chain is about 10 Å, which is sufficient to satisfy the requirement of the sensor–sample distance^36^. Compared with the conventional oxidation interface, the alkene-modified interface exhibits a notable reduction in interface states within the bandgap arising from the interface defects. This reduction is crucial in minimizing the undesirable influence on the operation of shallow qubits in SiC. In particular, the carbon-chain group at the interface demonstrates dynamic oscillatory behaviour at room temperature, reminiscent of water plants. These chains collectively form a hydrophobic layer, acting as a protective barrier against water molecules (Fig. 2b) and mitigating the corrosive impact on the SiC surface from the external environment, which contributes to the stable operation of the quantum sensor. On the other hand, the methyl groups at the end of the chain can be straightforwardly replaced with alcohol groups^37,38^, which makes the surface hydrophilic if required for a target bioapplication (Fig. 2c).

An additional key advantage arises from the negligible impact of carbon-chain termination of the surface on the electrical properties of the SiC (Fig. 2d). Specifically, compared with the SiC layer, the band edge shift of the valence band maximum (VBM) and conduction band minimum (CBM) in the carbon-chain layer is –0.03 and +0.03 eV, respectively. Consequently, the electrical properties of the SiC remain unperturbed, which is pivotal in ensuring the overall charge state stability and photostability of the shallow defect qubits. As an example, the magneto-optical parameters of the PL1 centre divacancy qubits in 4H-SiC with *S* = 1 electronic spin and zero-field splitting *D* = 1,336 MHz and *E* = 0 MHz electron spin resonance parameters in the ground state^6^ separating the *m*_s_ = 0 and other spin levels and the *m*_s_ = +1 and *m*_s_ = –1 levels, respectively, have remained almost unaltered within ~6 MHz when placed 1.3 nm below the interface (Supplementary Table I). In particular, we harness this divacancy species in 4H-SiC^6,7^, for which the controlled formation is well established^7^. Next, we characterize this quantum sensor in sensing applications with the optical read-out of electronic spin.

### Relaxometry sensing

**Fig. 3:**
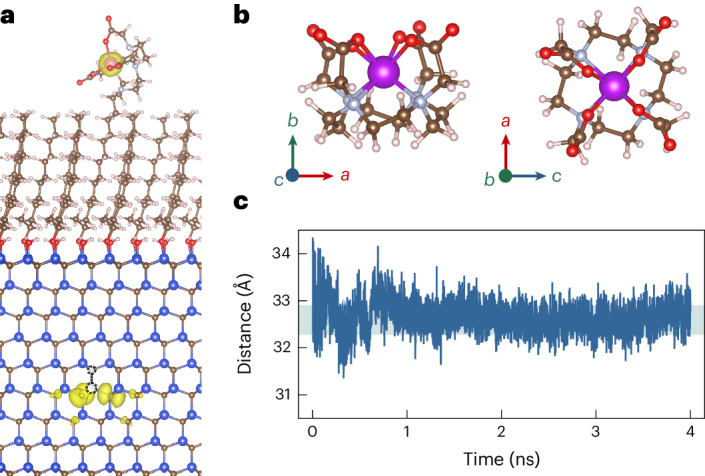
Spin interaction model between the Gd-DO3A complex and a near-surface divacancy in the carbon-chain interface. **a**, Spin density of the carbon-chain interface with the Gd-DO3A complex connecting to one of the carbon chains (isosurface value at 0.0025 electron Bohr^−3^). The divacancy resides 1.3 nm deep from the top surface. **b**, Atomistic picture of the Gd-DO3A complex. **c**, Distance between the Gd^3+^ ion and divacancy during the dynamic motion of atoms at room temperature. Source data

**Fig. 4:**
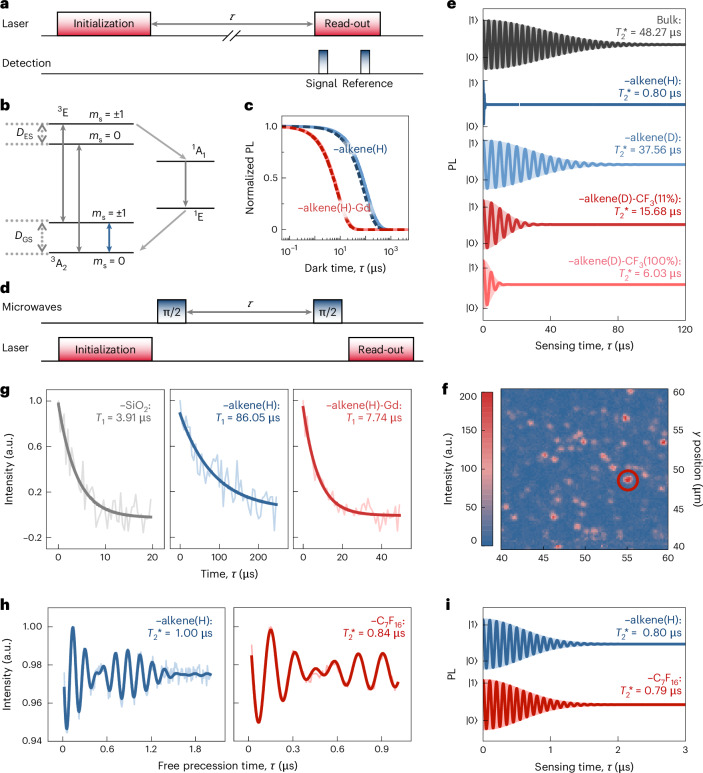
Spin relaxation and dephasing characteristics of near-surface divacancy quantum sensors in functionalized 4H-SiC. **a**, Pulse sequence used to measure the *T*_1_ time, with varying dark times *τ*. **b**, Energy-level diagram showing the ground state (GS) and excited state (ES) of the spin triplet (*S* = 1, left) and singlet (*S* = 0, right) for the divacancy quantum sensor. **c**, Simulated change in the *T*_1_ time of PL1 (solid) and PL6 (dash) divacancy centres under the fluctuating field of the Gd^3+^ spin. **d**, Ramsey sequence used to measure the ${T}_{2}^{* }$ spin dephasing time. **e**, Simulated Ramsey oscillation of ~1.3-nm deep single-divacancy PL centre in ^28^Si^12^C with deuterium-based alkene termination without and with replacing the methane group with the fluorine group at the end of the chains with the given percentage. **f**, Confocal scanning image of the near-surface PL6 centre. **g**, *T*_1_ times of the near-surface PL6 centre in SiC with the oxide surface and alkene(H) termination without and with the Gd-DO3A complex. **h**, Experimental observation of Ramsey oscillation for ~2-nm-deep single-divacancy PL6 centre in the naturally abundant SiC surface functionalized with alkene(H) and perfluoroheptane (C_7_F_16_). **i**, Theoretically calculated ${T}_{2}^{* }$ spin dephasing time of ~1.3-nm-deep single PL1 centre in the naturally abundant SiC surface with –alkene(H) and –C_7_F_16_ terminations. Source data

Relaxometry sensing—based on the observation of the *T*_1_ time of the quantum sensor—is one of the most successful applications of quantum sensors in biosensing^39^. To assess the sensitivity of 4H-SiC divacancy in detecting isolated spins, we positioned the Gd-DO3A complex with stable *S* = 7/2 spin on top of a carbon chain (Fig. 3a,b) to examine its impact on the *T*_1_ time of 4H-SiC divacancy’s spin. Gd-DO3A is a sophisticated Gd complex characterized by a well-designed molecular structure. At room temperature, the Gd-DO3A complex swings along with the carbon chain when attached to the alkene-terminated SiC surface. However, the motion of Gd-DO3A is fast enough to consider an averaged position of the Gd ion with respect to the position of the divacancy quantum sensor, which is about 3.2 nm (Fig. 3c). The quantum sensor is based on the optical read-out of the electronic spin of the divacancy, which can be realized by a confocal microscope setup when an antenna is engineered close to the area of investigation to induce alternating magnetic fields for electron spin resonance. The optical read-out is based on spin-dependent intersystem crossing towards intermediate singlet states. Optical pumping with a 914-nm laser of the quantum sensor leads to an efficient spin polarization into the *m*_s_ = 0 spin level, whereas the spin state can be read through spin-dependent PL as the intensity of the *m*_s_ = ±1 states is lower than the *m*_s_ = 0 state (Fig. 4b). The resulting state is detected by a relatively weak probe laser pulse at 914 nm where the read-out can be most efficiently carried out in the phonon sideband of emission in the region of 1.2–1.5 μm with the use of sensitive superconductor nanowire-based detectors. When no external electronic spins are present near the divacancy-based quantum sensors, the *T*_1_ time reaches 0.11 ms at room temperature (Supplementary Note 12), consistent with the result in ref. ^40^.

By positioning the Gd-DO3A complex at the top of the carbon chain at a distance of ~3.2 nm (Fig. 3a), we achieve almost two orders of magnitude reduction in *T*_1_, from 0.11 ms to 7.92 μs in our simulations (Fig. 4c and Supplementary Note 12). The result is comparable with that of the diamond NV centre^41^ but with the advantage of excitation wavelength optimal for biological applications. We conclude that the 4H-SiC divacancy quantum sensor with the alkene-terminated surface can be effectively utilized to detect paramagnetic molecules that can be straightforwardly attached to the end of the carbon chain by covalent bonds if required^32^.

### qNMR

The quantum sensor can also be effectively applied for qNMR measurements. In this case, it is useful to engineer the divacancy into ^28^Si^12^C-isotope-engineered host material (^29^Si and ^13^C abundances are about 0.15% and 0.02%, respectively^17^). Therefore, the spin dephasing time of the electron spin will be in the regime of 0.05 ms, as observed in the bulk region of 4H-SiC^17^. The divacancy spin situated at ~1.3 nm below the alkene-terminated SiC surface feels the proton spins attached to the carbon chains that are fluctuating at a much higher rate than the spin dephasing and spin relaxation times; thus, their effect on the probe divacancy spin can be spatially averaged of the position of the proton spins. The hyperfine coupling between the divacancy spin and proton spins is significant; therefore, ${T}_{2}^{* }$ reduces to ~0.80 μs (Fig. 4e). On the other hand, the quantum sensor is designed to sense external nuclear spins as small fluctuating magnets outside the protective carbon-chain layer. A straightforward avenue is to synthesize alkene molecules with deuterium and natural carbon isotope abundance; therefore, the bulk value of ${T}_{2}^{* }$ is almost retained (Fig. 4e). With these isotope-engineered materials, the magnetic field sensitivity reaches $\sim 13\,\,\text{nT}/\sqrt{\text{Hz}\,}$ for single-divacancy quantum sensor using pulsed electron spin resonance spectroscopy, which favourably competes with the best diamond-NV-based magnetometers^42^ with room-temperature optical read-out contrast of ${\mathcal{C}}=0.07$ (ref. ^7^) and ${T}_{2}^{* }\approx 38$ μs for a shallow PL1 centre. To illustrate this effect, a single methyl group at the end of the carbon chain is replaced by –CF_3_ groups. We note that the –CF_3_ groups do not introduce levels into the bandgap of 4H-SiC (Supplementary Fig. 7). The ${T}_{2}^{* }$ value is reduced by 73% due to the fluctuating magnetic field of the nuclear spins of fluorine atoms (Fig. 4e). When all the methyl groups are replaced by –CF_3_ groups via a nucleophilic substitution reaction^43–45^, then ${T}_{2}^{* }$ becomes ~6.06 μs. These ${T}_{2}^{* }$ values suffice to start the qNMR protocols (for example, XY8-N sequences) to observe the individual ^19^F spins and their couplings in the order of 10 kHz (refs. ^46,47^).

### Experimental results

We demonstrate the strength of prediction power and principles of simulations on near-surface divacancy species engineered into commercially available SiC samples with a natural abundance of nuclear spins (Methods). Although deuterium-based alkene chemistry is theoretically feasible, it is not yet practically available. Therefore, we proceeded with our experiments using a non-optimized SiC surface with natural alkene (–alkene(H)) termination. To this end, 4H-SiC samples were implanted with carbon ions at varying energies (1 keV and 5 keV) to create divacancy defects at controlled depths (2 nm and 7 nm), followed by annealing. The samples were then cleaned and coated with 1-alkene using a standard surface treatment procedure^32^. The corresponding experimental methods are detailed in the Methods and Supplementary Notes 10–12.

We studied single PL1 and PL6 centres in the these samples. The PL6 centre showed superior optical properties and continuous-wave ODMR read-out contrast (Supplementary Note 11); therefore, we proceeded with single PL6 centres that share the same symmetry and similar spin parameters with those of PL1 divacancies. In the naturally alkene-terminated SiC, the observed ${\mathcal{C}}\approx 0.1$ for a 2-nm-shallow PL6 centre is shorter than that reported for a deep PL6 centre (${\mathcal{C}}\approx 0.21$) at room temperature, but it is still favourable for quantum sensor applications. The origin of the reduced ${\mathcal{C}}$ for shallow PL6 centres is not yet clear. The *T*_1_ time of near-surface (~2-nm deep) PL6 centres reaches ~85 μs at room temperature, an order of magnitude longer than that of the same centres with an a-SiO_2_ layer present (Fig. 4g and Supplementary Fig. 18), and comparable with values reported for deeply buried divacancies in ref. ^40^. This demonstrates the predicted supremacy of 1-alkene termination over oxide termination. On further coating with Gd-DO3A, the *T*_1_ time drops to several microseconds (Fig. 4g and Supplementary Fig. 18), clearly demonstrating the impact of Gd^3+^-induced spin fluctuations on the relaxation dynamics of near-surface PL6 centres. As a representative example, the *T*_1_ time of one PL6 centre dropped from 86.05 μs to 7.74 μs after coating Gd-DO3A, showing one order of magnitude reduction (Fig. 4g). The result is in good agreement with our theoretical predictions and highlight the high sensitivity of shallow divacancy-related centres to local spin noise, establishing their potential for high-sensitivity quantum sensing applications. The experimental procedures for surface functionalization and *T*_1_ measurements are provided in the Methods and Supplementary Note 12.

Following the *T*_1_ relaxation measurements, we turned to examining the ${T}_{2}^{* }$ time to gain a deeper insight into the coherence dynamics of the spin system. The ${T}_{2}^{* }$ time of the 2-nm-deep PL6 centres is approximately 1 μs at room temperature (Supplementary Fig. 23). This is shorter than the bulk value in natural SiC in which the proton spins of the alkene chains reduce ${T}_{2}^{* }$ by about 0.5 μs. Nevertheless, this ${T}_{2}^{* }$ value is still long enough to detect proximate fluorine nuclear spins, replacing the proton spins. To investigate the influences of surface fluorine groups on the coherence properties, the surface is coated with perfluoroheptane (C_7_F_16_), following the protocol detailed in Supplementary Note 12. The ${T}_{2}^{* }$ time of the same PL6 centre decreased slightly to 0.84 μs after coating the C_7_F_16_ solution (Fig. 4h). This trend is consistent with our theoretical simulation (Fig. 4i), although the experimentally observed decrease is more pronounced, probably due to variations in the defect depth (~2 nm in the experiment and ~1.3 nm in the simulation). The anticipated sensitivity with the observed ${\mathcal{C}}$ and ${T}_{2}^{* }$ parameters approaches $56\,\,\text{nT}/\sqrt{\text{Hz}\,}$ in alkene-terminated natural SiC (Supplementary Note 8). These findings reflect the sensitivity of shallow divacancy centres to external nuclear spins—suggesting their potential for high-sensitivity qNMR measurements, which can be further improved when isotope-engineered SiC and alkene chains are used.

## Conclusion

In summary, we showed that the alkene-terminated 4H-SiC surface with shallow divacancy quantum sensors can be applied for electron spin relaxometry of radicals and other spin species using infrared excitation and emission for read-out in the second biological window at room temperature, making this system an excellent candidate for in vivo bioquantum sensor applications. Furthermore, the theoretical sensitivity of magnetometry for a single PL1 divacancy quantum sensor reaches $\sim 13\,\,\text{nT}/\sqrt{\text{Hz}\,}$ that may be used to sense nuclear spins at room temperature when the host material and alkene molecules are isotope engineered. We demonstrated with ultrashallow PL6 centres without any optimization in the isotope density and processing of the SiC surface that the room-temperature optical read-out contrast and coherence times make it possible to approach the same order of magnitude in the anticipated sensitivity. We note that the sensitivity can be enhanced by optimizing the optical read-out contrast of the divacancy-related centres by engineering the optical pulse schemes, as shown for the akin diamond NV centre^48^.

The flexibility of the alkene-terminated surface can be used not only for the functionalization of target bioquantum sensing applications but may be used to realize quantum simulation. We showed that the fluorine atoms at the end of the carbon chains can be observed by the divacancy quantum sensor. The bath of fluorine nuclear spins may realize a large-scale quantum simulator^49^ where the arrangement of the fluorine atoms depends on the temperature because of the swinging motion of the carbon chain (Supplementary Videos 1–3) in which the motion of the ions can be significantly slowed down and frozen into arranged positions at cryogenic temperatures. We envision that the transition of ordered and disordered quantum systems can be probed by our proposed system. In addition, this organic insulator may be applied as a non-conventional solution for improving the performance of SiC-based high-power optoelectronic devices, too.

## Methods

### Experimental methods

The material used in this work is a 30-μm-thick epitaxial layer of single-crystal 4H-SiC with a nitrogen-doping density of 1 × 10^14^ cm^−3^ grown on a 4° off-axis 4H-SiC substrate. The 4H-SiC samples were implanted with carbon ions at a dose of 1 × 10^11^ ions cm^−2^ and annealed at 900 °C for 30 min under a vacuum to generate divacancy defects. The implantation energies were 1 keV and 5 keV, resulting in central implantation depths of 2 nm and 7 nm, respectively (Supplementary Note 10).

After implantation, the samples were annealed in a vacuum at 900 °C for 30 min. The pressure during annealing is about 3 × 10^−4^ bar, which is not high enough to eliminate oxygen that might thermally oxidize the sample surface. After annealing, the samples were sonicated in acetone for 5 min and dried with nitrogen for cleaning. For the surface treatments, the sample was cleaned in a piranha solution at 90 °C in a water bath for 2 h, followed by immersion in GR-grade HF solution for 30 min to remove the insulating a-SiO_2_ layer. After rinsing with deionized water to remove the residual HF, the surface was immediately activated by sonication in alcohol. Subsequently, 1-alkene was dropped onto the surface and allowed to evaporate naturally.

### Measurement of *T*_1_

Relaxometry sensing—based on the observation of the *T*_1_ time of the quantum sensor—is one of the most successful applications of quantum sensors in biosensing^39^. To measure the *T*_1_ time of divacancy-related centres, we performed a standard inversion-recovery pulse sequence, in which a π pulse inverts the spin population, followed by a variable delay *τ* before a read-out pulse detects the spin state. All *T*_1_ times in this study were measured under 914-nm laser excitation at a power of 200 μW using a 0.85-numerical-aperture lens, with a static magnetic field of 180 Gauss. The relaxation time *T*_1_ is then extracted by fitting the decay of the PL signal using the following formula:

$$L(\tau )=A{\rm{e}}^{-\tau /{T}_{1}}\,\text{,}\,$$

where *A* and *T*_1_ are free parameters.

### Measurement of ${T}_{2}^{* }$

To quantify the coherence times, we first performed continuous ODMR and Rabi oscillation measurements to determine the resonant frequency and π pulse (representative data are provided in Supplementary Note 13). All ${T}_{2}^{* }$ results in this study were obtained under 914-nm laser excitation at a power of 200 μW using a 0.85-numerical-aperture lens, with a microwave power of –15 dBm and a static magnetic field of 180 Gauss. The microwave frequency detuning for the Ramsey sequences was set to +6 MHz. Details of the Ramsey pulse sequence and spin evolution on the Bloch sphere are provided in Supplementary Note 13. The experimental data were fitted using the formula

$$L(\tau )={{\rm{e}}}^{{(\tau /{T}_{2}^{* })}^{2}}[a\cos (2\pi {\delta }_{1}\tau +{\psi }_{1})\times b\cos (2\pi {\delta }_{2}\tau +{\psi }_{2})]+c,$$

where *a*, *b*, *c*, *ψ*_1_ and *ψ*_2_ are free parameters. The fitted detuning frequencies *δ*_1_ and *δ*_2_ closely matched the experimental settings. For single shallow PL1 centres, due to their low fluorescence intensity and ODMR contrast, it took an average of 3–4 days to measure ${T}_{2}^{* }$. By contrast, ${T}_{2}^{* }$ measurements for PL6 centres take only 3–4 h, owing to their high fluorescence intensity and ODMR contrast.

### Computational methodology

All the first-principles calculations are performed using density functional theory within the projector-augmented wave potential plane-wave method, as implemented in the Vienna ab initio simulation package (v. 5.4.1)^50^ with the projector-augmented wave method^51^. The electron wavefunctions are expanded in a plane-wave basis set limited by a cut-off of 420 eV. The fully relaxed geometries were obtained by minimizing the quantum mechanical forces between the ions falling below the threshold of 0.01 eV Å^−1^ and the self-consistent calculations are converged to 10^−5^ eV.

The screened hybrid density functional of Heyd, Scuseria and Ernzerhof (HSE06)^52^ is used to calculate the electronic structure. In this approach, we could mix part of the non-local Hartree–Fock exchange with the Perdew–Burke–Ernzerhof generalized gradient approximation^53^ at the default fraction (*α* = 0.25) and an inverse screening length at 0.2 Å^−1^. The single Γ-point scheme is convergent for the *k*-point sampling of the Brillouin zone. The hyperfine tensors for selected nuclear spins were calculated within projector-augmented wave formalism together with core polarization correction in the Fermi-contact hyperfine interaction, as implemented in the Vienna ab initio simulation package^54^. The excited states were calculated by the ΔSCF method^55^. We note that the reorganization energy and optimized geometry of the optically excited state can be calculated using the ΔSCF method, which are both important to predict the PL spectrum including the phonon sideband. For calculating the dipolar electron-spin–electron-spin *D* tensor, we use the implementation described in ref. ^56^ with the Perdew–Burke–Ernzerhof functional that successfully worked for the divacancy centres in 4H-SiC^57^.

### Oxidized interface model

The atomistic model of the 4H-SiC–a-SiO_2_ interface is created by simulating 4H-SiC oxidation with a-SiO_2_. The a-SiO_2_ layer is first prepared by simulating the melting and quenching of α-quartz SiO_2_ (a polymorph of crystalline SiO_2_) using classical molecular dynamics (MD) with the ReaxFF reactive force field implemented in the large-scale atomic/molecular massively parallel simulator code^58–62^. An orthorhombic supercell of the interface is created by stacking a-SiO_2_ on top of the Si-terminated (0001) surface of 4H-SiC. The lateral dimensions correspond to the $\sqrt{16}\times \sqrt{12}$ SiC(0001) surface unit cell, and there are eight Si–C bilayers in the SiC side with a thickness of about 20.26 Å. The thicknesses of the a-SiO_2_ and vacuum layers are 14.83 Å and 15.00 Å, respectively. There is a 2-Å gap between the a-SiO_2_ and crystalline-SiC slab.

The oxidation of 4H-SiC to a-SiO_2_ is simulated by classical MD with ReaxFF at a pressure of 1 atm and oxidation temperatures of 1,000–1,500 K. The Berendsen thermostat and barostat are applied to simulate an isothermal–isobaric ensemble^63^. The supercell is heated up from 0 K to the oxidation temperature in 15 ps, maintained at that temperature for 200 ps and then cooled down to 0 K in 50 ps. The time step of the MD simulations is 0.5 fs. The few point defects generated in the a-SiO_2_ side in oxidation, such as oxygen vacancies and interstitials, are removed manually, and the dangling bonds on the upper and lower surfaces of the models are saturated by hydrogen atoms. The models are then optimized using first-principles calculations by the Vienna ab initio simulation package code. We embedded the interface defects in the 4H-SiC–a-SiO_2_ interface model, with lateral dimensions of 10.72 × 12.38 Å^2^, which is sufficient to minimize the periodic defect–defect interaction.

### Carbon-chain interface model

The atomistic model of the 4H-SiC–alkene chain is directly taken from the experimental data^32^. The Si-terminated (0001) surface of 4H-SiC was first terminated by –OH groups. Then, alkene chains (CH_2_=CHC_11_H_23_) were added by creating an oxygen bridge between the SiC surface and the alkene chain in the densest form. The lateral dimensions correspond to the 6 × 6 SiC(0001) surface unit cell, and there are twelve Si–C bilayers in the SiC side with a thickness of about 28.45 Å. The bottom SiC layer was terminated by hydrogen, to eliminate the dangling bonds. The PL1 centres (*hh* divacancies) were placed near the interface.

We realized that an electric field develops between the alkene-terminated top layer and hydrogen-terminated bottom layer due to charge transfer between the terminator species and the SiC layers together with the in-built electric field in the 4H-SiC crystal caused by differently polarized *h* and *k* bilayers in the 4H-SiC crystal^64^. In experiments, these electric fields are compensated by attracting ionic-like species to the surface of a macroscopic SiC in the bottom and top layers. Instead of atomic simulations of this process, we rather applied a compensating electric field along the *c* axis, which is 0.25 V Å^−1^ in our particular slab model. We find that the application of this compensating field is critical in the accurate calculation of the *D* tensor of the PL1 centre near the interface because of its coupling to the electric field^57^.

### Reporting summary

Further information on research design is available in the Nature Portfolio Reporting Summary linked to this article.

## Online content

Any methods, additional references, Nature Portfolio reporting summaries, source data, extended data, supplementary information, acknowledgements, peer review information; details of author contributions and competing interests; and statements of data and code availability are available at 10.1038/s41563-025-02382-9.

## Supplementary information

Supplementary Information Supplementary Notes 1–13, Tables I–IV and Figs. 1–25.

Reporting Summary

Supplementary Video 1 Swinging motion of the carbon chain with the methyl group at the end of the carbon chain and a water droplet on top.

Supplementary Video 2 Swinging motion of the carbon chain with the alcohol group at the end of the carbon chain and a water droplet on top.

Supplementary Video 3 Swinging motion of the carbon chain with a Gd cluster.

## Source data

Source Data Fig. 1 Source data for Fig. 1.

Source Data Fig. 2 Source data for Fig. 2.

Source Data Fig. 3 Source data for Fig. 3.

Source Data Fig. 4 Source data for Fig. 4.

## Data Availability

All data used for this study are available within the Letter and its Supplementary Information. Source data are provided with this paper. The data files are also available from the corresponding authors upon request.
