# Supplementary material for: Non-invasive bioinert room-temperature quantum sensor from silicon carbide qubits
Source: Nat Mater. 2025 Nov 6;24(12):1913–9. doi: 10.1038/s41563-025-02382-9 (PMC12657224; doi:10.1038/s41563-025-02382-9)
Supplement: Supplementary file 1 — Supplementary Notes 1–13, Tables I–IV and Figs. 1–25. [file 41563_2025_2382_MOESM1_ESM.pdf]

nature materials

Supplementary information

https://doi.org/10.1038/s41563-025-02382-9

Non-invasive bioinert room-temperature quantum sensor from silicon carbide qubits

In the format provided by the authors and unedited

CONTENTS

Supplementary Note 1: Defects at the 4H-SiC/a-SiO_2 interface 2

Electronic structure of interface defects 2

Optical spectrum of interface defects 5

The carbon cluster consisting of four carbon atoms 6

Supplementary Note 2: Divacancy quantum sensors near the surface of alkene-terminated 4H-SiC 6

Supplementary Note 3: The carbon-chain interface 8

Supplementary Note 4: Electronic structure of $-CF_3$ terminated carbon-chains 9

Supplementary Note 5: Spin noise sensing by observation of the spin-lattice relaxation time 10

Supplementary Note 6: Electron spin coherence time 12

Supplementary Note 7: Quantum sensing 13

Supplementary Note 8: Magnetic field sensitivity 15

Supplementary Note 9: Experimental setups 16

Supplementary Note 10: Confocal scanning images for SiC samples with divacancies. 16

Supplementary Note 11: ODMR and Rabi oscillation 19

Supplementary Note 12: Spin-lattice relaxation time: T_1 20

Supplementary Note 13: Transverse relaxation time: T_2^* 23

References 28

SUPPLEMENTARY NOTE 1: DEFECTS AT THE 4H-SIC/A-SIO$_2$ INTERFACE

In this study, we focused our investigations on the origin of photoluminescence (PL) centres and quantum emitters at the 4H-SiC/a-SiO$_2$ interface. The interface model was created as described in the Method section of the main text. There we intentionally removed defect species spontaneously formed in the molecular dynamics simulation, in order to obtain a so-called "ideal" abrupt interface between SiC and the amorphous oxide. This approach makes it possible to analyse this "ideal" structure and how the defects alter their optoelectronic properties.

The typical carbon cluster models were taken from previous models [1–5] at the interface and from our recent calculations in the bulk SiC [6]. We focused on finding the origin of the quantum emitters by comparing the published PL spectrum and simulated ones, thus we were motivated to find such carbon clusters that might have local vibration modes in the respective region. Besides carbon clusters, we also considered other defects such as Si dangling bonds, Si-Si dimers, and oxygen-related defects (around 100 configurations). The atomistic structures of selected defects are shown in Supplementary Fig. 1, and the corresponding electronic structures in the ground state can be found in Supplementary Fig. 2.

Electronic structure of interface defects

During the oxidation process, the SiC surface oxidation process initiates as oxygen (O) atoms adsorb onto the SiC surface. Initially, weak physisorption or stronger chemisorption may occur, depending on the surface conditions [7]. Chemisorption leads to the formation of robust Si−O and C−O bonds. Following adsorption, O atoms can diffuse across the SiC surface, due to their affinity for Si and C atoms, forming the O interstitial defects [see Supplementary Figs. 1(a) and (b)]. The O interstitials (O_i) are in singlet spin state and there are two occupied defect levels (DLs) near the valence band maximum (VBM) [see Supplementary Figs. 2(a) and (b)]. Once the silicon dioxide layer attains a certain thickness, Si-Si defects [see Supplementary Fig. 1(g)] may emerge due to the absence of O atoms in SiO$_2$ layer, akin to the dimer defects observed in SiO$_2$ [8]. Concurrently, as carbon atoms escape from SiC, they may also give rise to analogous dimer defects [see Supplementary Fig. 1(f)]. Dimer defects comprising C or Si atoms exhibit sp^3 hybridization, akin to the defect exemplified in Supplementary Figs. 1(p) and (q). Typically, DLs are not discernible within the band gap for these defects [see Supplementary Figs. 2(f) and (q)]. However, under the influence of twisting induced by surrounding structural stress, the resulting local geometry may engender the introduction of shallow DLs in close proximity to the VBM within the band gap, as elucidated in Supplementary Figs. 2(g) and (p).

For C-related defects forming sp^2 and sp^3 hybridization, such as C=C in Supplementary Figs. 1(c)-(e), there are two occupied and two unoccupied DLs. As the defect transitions from the SiC layer to the SiO$_2$ layer, a notable trend emerges: the DLs progressively approach the band edge, as visually represented in Supplementary Figs. 2(c)-(e). On the contrary, for carbon interstitial defects, which also exhibit sp^2 hybridization [see Supplementary Figs. 1(m) and (r)], the DL behavior is distinct. In the SiO$_2$ layer, where the carbon interstitial bonds with three O atoms, two deep occupied DLs emerge within the band gap, as depicted in Supplementary Fig. 2(m). Conversely, when the carbon interstitial resides in the SiC layer, the defect assumes a high-spin configuration, resulting in one occupied spin-up DL and one unoccupied spin-down DL [see Supplementary Fig. 2(r)]. When the carbon interstitial is squeezed into

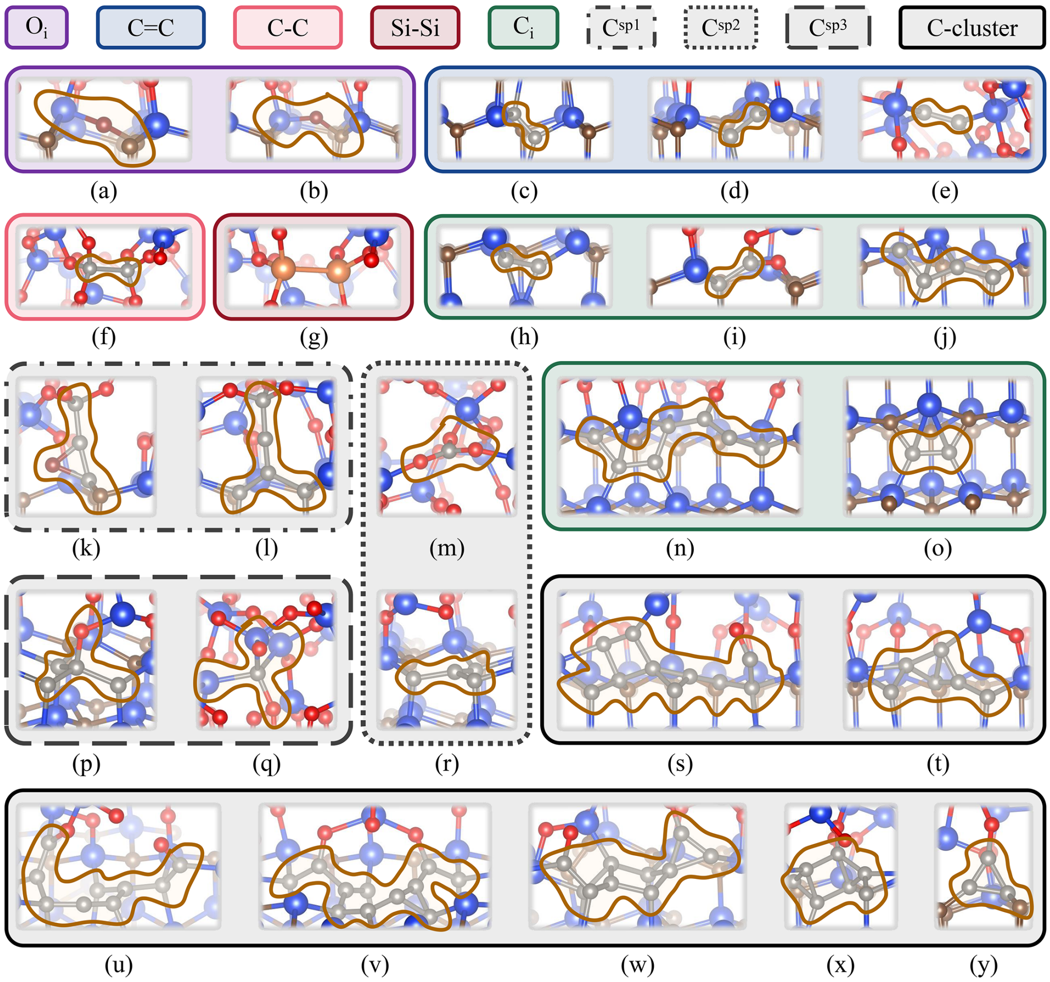

Supplementary Fig. 1. The local geometry of the interface defects. Various types of defects are differentiated by distinct colours and styles of boxes, with corresponding defect labels positioned at the top.

a narrow space, localized stress can cause structural distortion of the carbon interstitial, as shown in Supplementary Figs. 1(h) and (i). In such cases, unoccupied defect levels within the band gap may move into the conduction band, leaving two occupied defect levels, as seen in Supplementary Figs. 2(h) and (i). When two carbon interstitials converge to form a defect pair [see Supplementary Figs. 1(j), (n) and (o)], the electronic structure of the defect is intricately tied to its local environment. Specifically, when the defect pair is situated within the interface transition region, characterized by a more diverse array of surrounding structures, it typically gives rise to two occupied DLs near the VBM within the band gap [see Supplementary Fig. 2(j)]. Nevertheless, when the defect pair is positioned within the SiC layer, the structural symmetry of the defect pair is enhanced, exhibiting characteristics more consistent with sp^2 hybridization [see Supplementary Figs. 1(n) and (o)]. Consequently, the defect typically introduces two occupied and two unoccupied DLs within the band gap [see Supplementary Figs. 2(n) and (o)].

In addition to sp^2 hybridization, the carbon interstitial can also form sp^1 hybridization, as depicted in Supplementary Figs. 1(k) and (l). These defects are typically located in regions characterized by low atomic density, where carbon interstitials bond two neighboring atoms, thereby forming a linear structure. The neighboring atoms, along

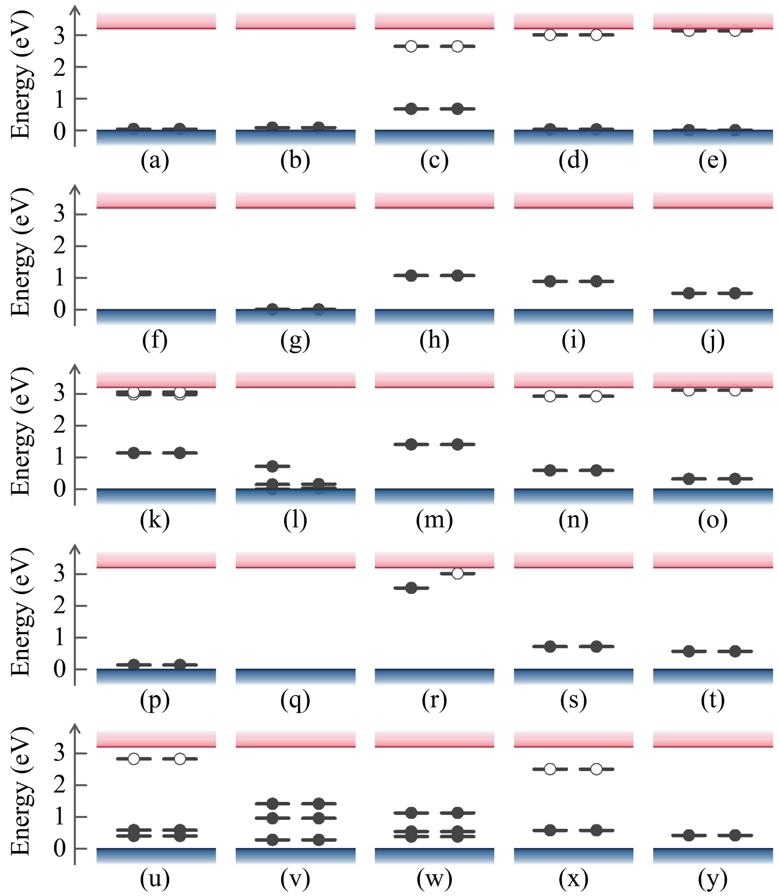

Supplementary Fig. 2. Calculated Kohn-Sham defect levels of the interface defects shown in Supplementary Fig. 1 in the fundamental band gap of the carbon-chain interface. The occupied and unoccupied defect levels are labeled by filled and empty circles, respectively.

with surrounding atoms, adopt either a planar or tetrahedral arrangement. The intrinsic flexibility of linear structures renders defect geometry highly sensitive to variations in the surrounding environment, a characteristic that is also evident in the electrical properties of the defects [see Supplementary Figs. 2(k) and (l)].

Aside from the previously mentioned defects, there are more complex defects present in the interface transition region known as carbon clusters (C-clusters) [see Supplementary Figs. 1(s)-(y)]. These defects arise from the accumulation of carbon atoms in the interface transition region during the later stage of the SiC oxidation process [7, 9, 10]. Carbon clusters typically consist of multiple carbon atoms, each with varying hybridization. As a result, the electrical behavior of these defects demonstrates significant diversity [see Supplementary Figs. 2(s)-(y)]. The most stable defects produce a fully occupied defect level in the band gap close to the VBM which agrees well with our recent findings in the bulk region of 4H-SiC [6].

Optical spectrum of interface defects

To identify the PL centres of the 4H-SiC/a-SiO_2 interface in experiments [11], we calculated the optical properties of the interface defects. In the calculation of the vertical excitation energies, the geometries were fixed at the ground state. For zero-phonon-lines (ZPLs) energies, the atoms are allowed to relax for the given electronic structure where the excited states were calculated by ΔSCF method [12]. The results of the vertical excitation energies and ZPLs are shown in Fig. 1 in the main text. The defect emission is a radiative decay from the bound exciton state for most of the carbon clusters apart from few exceptions. The bound exciton excited state has supercell size dependence, thus the accuracy of these energies is estimated to be within 0.2 eV which is larger than the anticipated accuracy for the defect-level to defect-level excitation within the band gap (see Ref. 13 and references therein).

Subsequently, we simulated the PL spectrum for these interface defects. For the vibrational modes, we calculated the corresponding dynamical matrix containing the second-order derivatives of the total energy by means of the Perdew, Burke, and Ernzerhof (PBE) functional [14]. The total Huang-Rhys factor (HR factor) is calculated within the Franck-Condon approximation which assumes that the vibrational modes in the ground and excited states are identical. The associated phonon overlap spectral function can be derived from the overlap between the phonon modes in the electronic ground and excited states [12, 15].

The results of local vibration modes (LVMs) are depicted in Fig. 1 in the main text. The interface defects exhibit distinct high-energy LVMs ranging between 120 and 200 meV. Additionally, many of the carbon-related defects under investigation display high-energy modes (see Fig. 1 in the main text) which occur due to short carbon-carbon bonds as we described in Ref. 6. Supplementary Fig. 3 illustrates the PL spectra of di-carbon antisite [$(2C)_{Si}$] and tri-carbon antisite [$(3C)_{Si}$] defects at various positions along the interface, serving as representative examples.

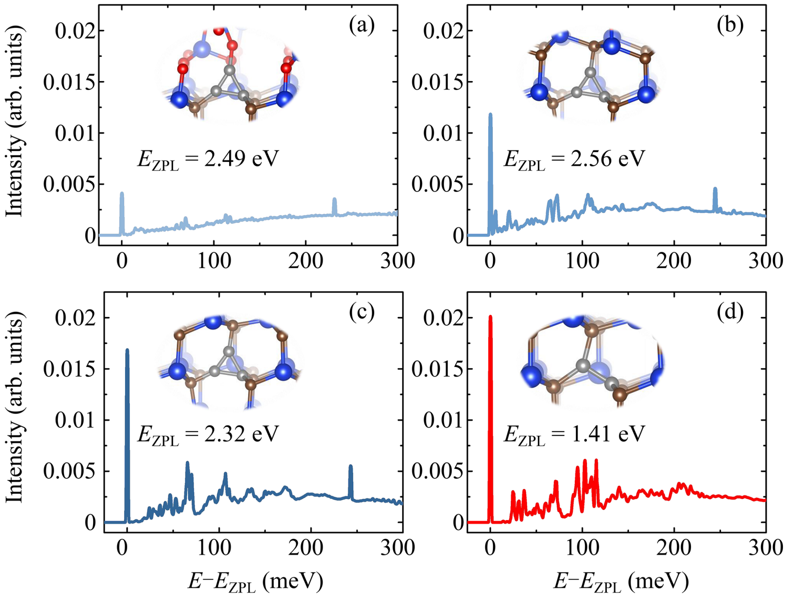

Supplementary Fig. 3. PL spectra of (a)-(c) tri-carbon antisite [$(3C)_{Si}$] and (d) di-carbon antisite [$(2C)_{Si}$] defects. Insert: the configurations of $(3C)_{Si}$ and $(2C)_{Si}$. The values of the ZPL are 2.49, 2.56, 2.32 and 1.41 eV, respectively.

When $(3C)_{Si}$ resides in the interface transition region [see Supplementary Fig. 3(a)], the minimum C−C bond length is 1.314 Å, with the highest LVM reaching approximately 230 meV. Due to its proximity to the a-SiO_2 layer, the surrounding atoms' types and structures are relatively complex. Consequently, significant structural changes occur in the defect during electron excitation, resulting in a relatively high HR factor of 4.6. If $(3C)_{Si}$ is in the SiC layer, but near the transition region [see Supplementary Fig. 3(b)], the length of the C−C bond diminishes to 1.298 Å, corresponding to the highest LVM of approximately 250 meV. As $(3C)_{Si}$ predominantly resides within the SiC layer, the atomic structure is more orderly compared to the transition region. Consequently, the relatively minor structural changes induced by electron excitation reduce the HR factor to 3.6. For $(3C)_{Si}$ within the SiC layer [see Supplementary Fig. 3(c)], the corresponding bond length, highest LVM, and HR factor are 1.293 Å, 250 meV, and 3.3, respectively. It is evident that defects situated in the interface transition region undergo relatively significant structural changes during electron excitation, resulting in a higher HR factor. Additionally, shorter C−C bonds in carbon clusters correspond to higher energy of the highest LVM.

To provide additional insight into the relationship between bond length and the highest LVM, we conducted an analysis of $(2C)_{Si}$ situated within the SiC layer [see Supplementary Fig. 3(d)]. The C−C bond length of $(2C)_{Si}$ is 1.416 Å, corresponding to the highest LVM of approximately 210 meV. This analysis further confirms that short C−C bonds contribute to high-energy LVMs, by the comparison with the results of $(3C)_{Si}$.

The carbon cluster consisting of four carbon atoms

As shown in Fig. 1 in the main text, the carbon cluster consisting of four carbon atoms produces a very similar PL spectrum to the observed one with a ZPL peak at around 2.1 eV [11]. The cluster is composed of four adjacent carbon split interstitials that are inclined towards each other, forming a tetrahedral ring [see Supplementary Fig. 4(a)]. The value of the ZPL is 2.27 eV and the highest LVM is approximately 225 meV [see Supplementary Fig. 4(b)]. The highest energy LVM at 187.72 meV contributes but not the LVMs ranging between 210 and 225 meV to the optical excitation process [see Supplementary Fig. 4(c)]. However, the higher order phonon replica of $\sim$ 110-meV LVMs produce visible peaks around 225 meV in the phonon sideband.

Due to the cluster proximity to the a-SiO_2 layer, significant structural changes occur in the defect during electron excitation, resulting in a relatively high HR factor of 6.58. The high HR factor implies that a small fraction of the optical transition is emitted as a zero-phonon line emission; most of the emission is rather distributed in the phonon sidebands [see Supplementary Fig. 4(b)].

SUPPLEMENTARY NOTE 2: DIVACANCY QUANTUM SENSORS NEAR THE SURFACE OF ALKENE-TERMINATED 4H-SIC

We considered the so-called PL1 centre [16–18], i.e., adjoint silicon-vacancy and carbon-vacancy in the hexagonal lattice site of 4H-SiC. Divacancies in SiC possess several unique characteristics that make them particularly attractive for quantum sensing, compared to silicon vacancies, which have also been demonstrated as quantum sensors. One of the key differences is that divacancies are neutral which does not require any dopants near divacancy qubits [19].

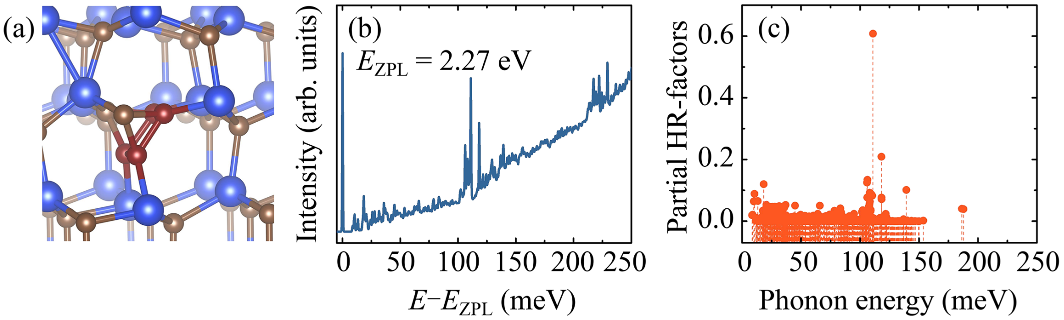

Supplementary Fig. 4. (a) The local configuration, (b) PL spectrum, and (c) partial HR factor for carbon clusters consisting of four carbon atoms.

This is not the case for silicon vacancy qubits which require donors to realize negatively charged silicon vacancies as qubits. So defect engineering of silicon vacancies requires to engineer both the silicon vacancy and its donor partner (typically, substitutional nitrogen) [20]. Additionally, divacancies in SiC are more thermally stable and can operate in harsher environments, including high temperatures, without significant degradation of their quantum properties [21]. According to reported measurements under similar conditions, divacancies offer superior stability [22], similar coherence times ($T_2^* \approx 30 - 50$ μs in isotope engineered SiC) [23–25] but significantly higher ODMR contrasts ($10 - 20\%$ vs. 0.5%) and saturating photon counts ($\sim$150 vs. $\sim$10 kcps) [26, 27], and robustness in harsh environments [21], compared to the silicon vacancy. Based on the standard sensitivity formula towards magnetic fields in Refs [28–30], the divacancy-based quantum sensors therefore offer at least an order of magnitude higher sensitivity, as was demonstrated [31–36].

PL1 centre has C_{3v} symmetry in the bulk region of 4H-SiC with a very similar electronic structure to that of diamond nitrogen-vacancy (NV) centre except that an additional empty double degenerate e-level appears close to the conduction band minimum (see Fig. 2 in the main text). However, that e-level does not participate in the optical excitation of the defect and can be ignored in the context. The observed optically detected magnetic resonance readout contrast $\mathcal{C} = 7\%$ at room temperature for a single PL1 divacancy defect falls in orders of magnitude into that of diamond NV centre [26]. In our study, we picked up this divacancy configuration in 4H-SiC for the sake of simplicity in terms of the demonstration of the SiC based quantum sensing: (a) the structure of PL1 is well-identified [17, 18]; (b) controlled formation of PL1 centres is well documented [37–39]; (c) it has favourable room temperature properties; (d) the similarities in the electronic structures of PL1 centre and diamond NV centre makes it viable to directly apply those techniques to PL1 divacancy configuration that were already proven for diamond NV centre in various sensing procedures. We note that the so-called PL6 centre [16, 40] has improved room-temperature $\mathcal{C}$ value [26] which can lead to improved sensitivity over that of the considered PL1 centre. However, the controlled formation of PL6 centres is still under intense research so we restrict the theoretical study to the well-established PL1 centre.

We first calculated the bulk value in a 576-atom supercell model of 4H-SiC with using the Perdew-Burke-Ernzerhof (PBE) functional [14] which resulted in $D = 1433$ MHz that is close to the experimental data at $D = 1336$ MHz [37]. We use the simulated bulk value as a reference to the simulations or PL1 centres that are placed at 2.53 Å, 7.59 Å,

and 12.66 Å deep from the top surface of alkene-terminated 4H-SiC. The Si-vacancy part of the defect was used as a reference point to measure the distance because the electron spin density is localized in the Si-vacancy part of the defect at a large extent [41]. The presence of the surface breaks the C_{3v} crystal field of 4H-SiC. As the $S = 1$ electron spin is very sensitive to small crystal field perturbations, the lowering of the C_{3v} crystal field is manifested in the presence of the splitting of the $m_S = +1$ and $m_S = -1$ spin levels in the electronic ground state at zero external magnetic field that is the so-called E orthorhombic parameter in the electron spin resonance spectroscopy. We find that this E parameter fast converges to the bulk value as a function of distance from the surface (see Supplementary Table I) where the bulk-like region in our model lies close to the middle of the slab model (about 1.3 nm deep from the surfaces).

The result for the 12.66 Å deep divacancy defect spin is almost identical to the bulk value. The origin of this effect is rooted in the strain field that is generated by the surface termination which rather causes a little non-symmetrical distortion of the ions around the Si-vacancy but more relaxation of ions along the c-axis of the 4H-SiC. This is reflected in the variation of the D axial parameter of the zero-field splitting where the deepest defect's D parameter practically agrees with the bulk reference value as obtained in our PBE simulation. This demonstrates that 1.3-nm-deep PL1 divacancy quantum sensor ground state magneto-optical properties are practically identical to the bulk values, thus the suggested surface termination does not introduce significant perturbation to the quantum sensor.

We note that the PL1 centre located nearest to the interface already have oxygen bonds in the third neighbour shell (see Supplementary Fig. 5) thus the spin density is directly affected by the interface and not only through the strain field. As a consequence, the D parameter of that centre does not fit to the usual trend that the strain field somewhat increases the D parameter for near-surface PL1 centres when compared to that of the bulk value.

Supplementary Table I. The changes of the zero-field splitting parameters for the PL1 divacancy centre for alkene-terminated surface. The experimental values for the bulk 4H-SiC region are $D = 1336$ MHz and $E = 0$ MHz [37].

distance (Å) from surface	D (MHz)	E (MHz)
2.53	−34.51	+29.11
7.59	+39.32	+7.16
12.66	+6.86	+3.47

SUPPLEMENTARY NOTE 3: THE CARBON-CHAIN INTERFACE

In the experiment, the interface can be achieved as follows [42, 43] [see Supplementary Fig. 6(a)]. First, the SiC wafer is rinsed multiple times with acetone, sonicated in acetone for 10 minutes, and dried with argon gas. It is then cleaned using air plasma for 10 minutes. Next, the SiC substrates are etched in a 2.5% aqueous hydrogen fluoride (HF) solution for 2 minutes, thoroughly rinsed with Milli-Q water, and dried with argon. The etched samples can then be transferred to a three-necked flask containing 2 mL of alkene-terminated monolayers. The flask is purged with argon under reduced pressure (10 mbar) for 30 minutes while heating to 80°C, followed by a reaction at 130°C for 16 hours. Finally, the samples are removed, rinsed with pentane and DCM, sonicated in DCM for 5 minutes to

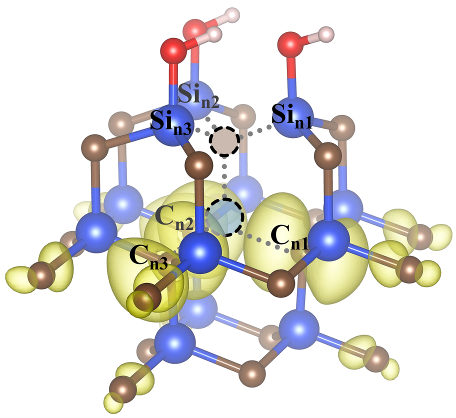

Supplementary Fig. 5. The local geometry of the divacancy in the nearest hexagonal layer to the interface. C_{n1}-C_{n3}, Si_{n1}-Si_{n3} are the first nearest neighbors to the divacancy. Isosurface value is set to 0.002 electron/bohr^3.

remove adsorbed molecules, and dried with argon.

The geometry of the interface is shown in Supplementary Fig. 6(b), highlighting the arrangement of atoms and structural configuration involved in the computational model, which serves as the foundation for the analysis and results. The lateral dimensions correspond to the 6×6 SiC(0001) surface unit cell, and there are twelve Si-C bilayers in the SiC side with a thickness of about 28.45 Å. The bottom SiC layer was terminated by hydrogen, in order to eliminate the dangling bonds. The PL1 centres (*hh* divacancies) were placed near the interface.

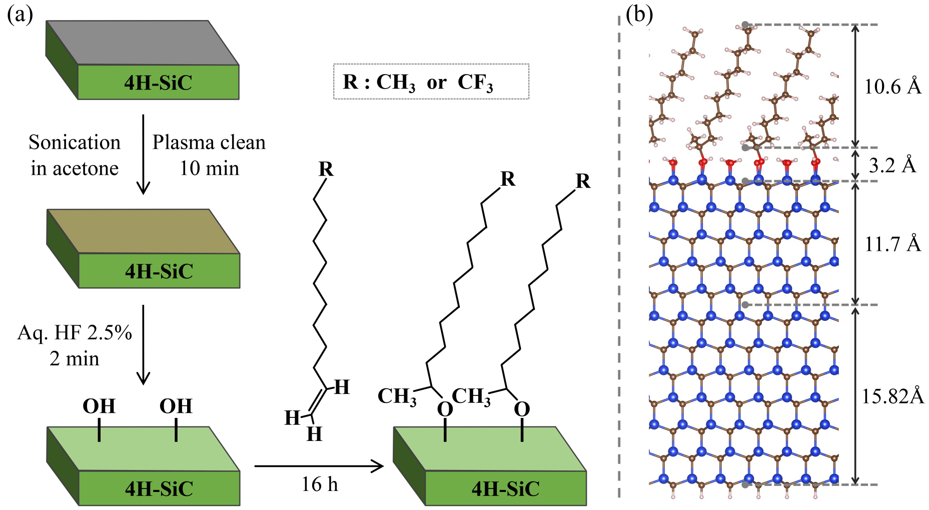

Supplementary Fig. 6. The legend of (a) passivation and (b) geometry of the carbon chain interface model.

SUPPLEMENTARY NOTE 4: ELECTRONIC STRUCTURE OF $-CF_3$ TERMINATED CARBON-CHAINS

Here we plot the electronic structure of the alkene-terminated 4H-SiC when a single methyl group is replaced by $-CF_3$ group or all the methyl-groups are replaced by $-CF_3$ group (Supplementary Fig. 7). We find that no surface

state appears in the fundamental band gap of 4H-SiC. The band gap of the perfect alkene-terminated 4H-SiC is 3.05 eV, which is smaller than the value of 4H-SiC bulk (3.17 eV) because of the potential shift between the upper and lower Si-C bilayer (Supplementary Fig. 8). If a single methyl-group is replaced by $-CF_3$ group, the band gap is 3.06 eV and the PDOS (Projected Density of State, PDOS) of fluorine atoms is in valence band ($E_V - 5.67$ eV), which means the $-CF_3$ group does not affect the band gap of alkene-terminated 4H-SiC. When all the methyl groups are substituted with $-CF_3$ group, the potential difference decreases (Supplementary Fig. 8) resulting in the band gap of 3.15 eV, which closely approximates the band gap of bulk SiC.

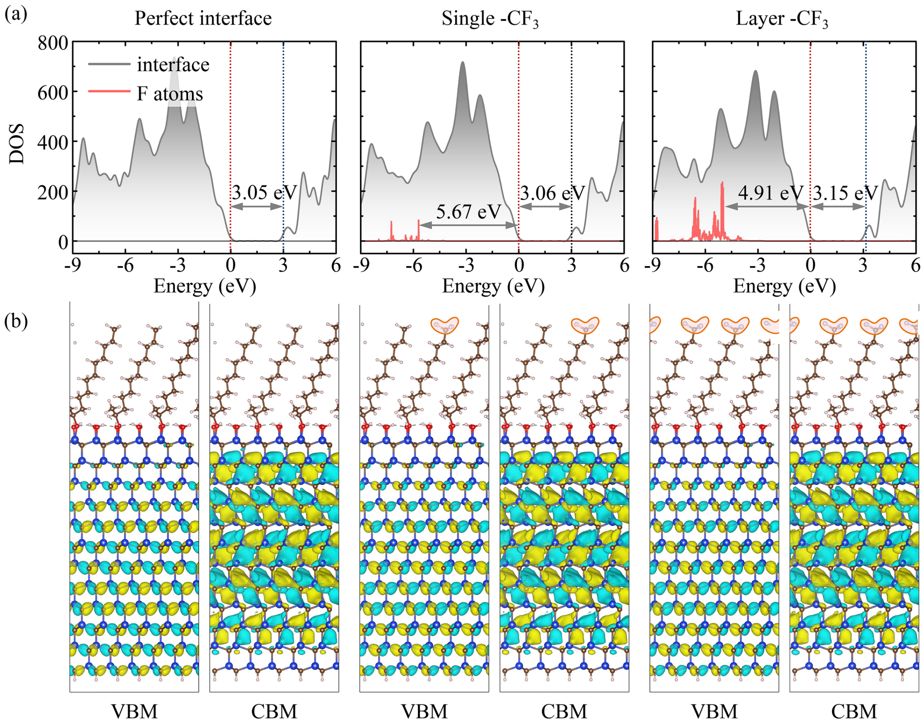

Supplementary Fig. 7. The electronic structure of alkene-terminated 4H-SiC when it is perfect, a single methyl group is replaced or all the methyl groups are replaced by $-CF_3$ groups. (a): the density of states (DOS). (b): the wavefunction of valence band maximum (VBM) and conduction band minimum (CBM) with an external electric field of 0.25 V/Å in order to compensate for the artificial electric field along the c-axis. Isosurface value is set to 1.2×10^{-5} electron/bohr^3.

SUPPLEMENTARY NOTE 5: SPIN NOISE SENSING BY OBSERVATION OF THE SPIN-LATTICE RELAXATION TIME

As a demonstration of the sensitivity of the quantum sensor for nearby radicals, we observed the effect of Gd-DO3A complex with $S = 7/2$ spin when placed near the quantum sensor engineered 1.266 nm deep from the surface of alkene-terminated 4H-SiC. We note that the approximate length of the alkene group is about 2.095 nm, thus the average distance between the observed Gd-DO3A complex and the quantum sensor is about 3.36 nm. We find that the majority of the spin densities are well localized around the Gd-ion and the Si-vacancy part of the PL1 centre (see Fig. 3 in the main text), so one can apply the electronic spin-spin point-dipole–point-dipole model in the analysis

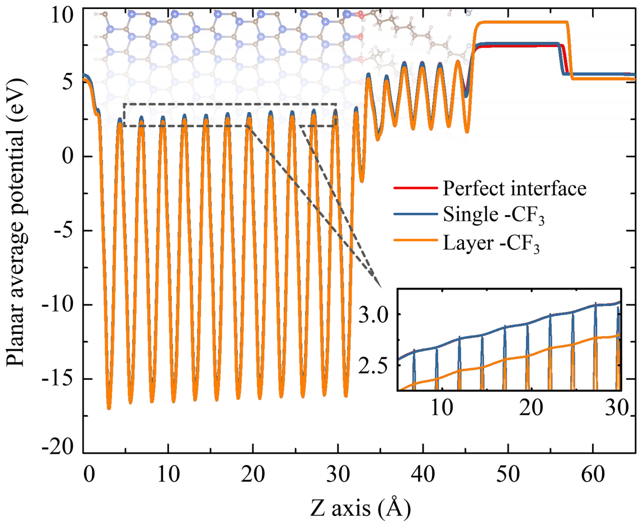

Supplementary Fig. 8. The planar average potential along the axis when it is perfect, a single methyl group is replaced or all the methyl groups are replaced by $-CF_3$ groups.

of the interaction between the quantum sensor and the Gd-DO3A spin label. If the quantum sensor is placed right to the interface then the spin densities somewhat overlap but this extreme situation is not likely to occur in proper defect engineering of the PL1 quantum sensor.

The longitudinal spin relaxation time, T_1, refers to the time it takes for the spin system to return to its equilibrium state in the direction of an external magnetic field after being perturbed from that state [44]. In the context of quantum sensing with T_1 time is a critical parameter. It influences the sensitivity of the sensor and is employed in various applications [45–47]. Gadolinium, Gd^{3+}, ion are intriguing spin systems due to their substantial unpaired electron spin ($S = 7/2$) and rapid spin dynamics in the gigahertz frequency range. These characteristics render Gd compounds highly effective as magnetic resonance imaging (MRI) contrast agents [48], where the presence of Gd reduces the relaxation time of protons in water. Remarkably, the Gd spin noise also affects the NV spin-relaxation time [49, 50]. In this section, we investigate the spin sensitivity of single Gd by the variation of T_1 time of divacancy in SiC with the surface being passivated by carbon chains.

In our simulation, the Gd-DO3A complex is positioned on the top of a carbon chain. The T_1 time of divacancy is reduced under the substantial magnetic moment from the Gd-DO3A complex ion according to the expression [45, 49]

$$\frac{1}{T_1} = \frac{1}{T_{1,int}} + \frac{3\gamma^2\tau_c}{1+\omega_{\mathrm{D}}^2\tau_c^2}\mathbf{B}_\perp(\mathbf{r})^2, \tag{1}$$

where the first term on the right side of the equation denotes the intrinsic T_1 time of divacancy in SiC (0.11 ms at room temperature, see Supplementary Note 12) and the second term signifies the additional relaxation rate due to Gd-DO3A complex. $\gamma = 2\pi \times 28$ GHz/T is the electron gyromagnetic ratio. $\tau_c = 0.36$ ns is the effective correction time of the Gd noise spectrum. $\omega_{\mathrm{D}} = 2\pi \times D$, in which $D = 1.336$ GHz is the energy level splitting between the $m_{\mathrm{s}} = 0$ and $m_{\mathrm{s}} = \pm 1$ states at zero external magnetic field. $\mathbf{B}_\perp(\mathbf{r})$ is the perpendicular magnetic field the divacancy

experiences from the Gd at position $\mathbf{r}$, which is given by

$$\mathbf{B}(\mathbf{r}) = \frac{\mu_0}{4\pi}\frac{g_{\mathrm{Gd}}\mu_B}{|\mathbf{r}|^3}\left[\mathbf{S} - \frac{3\mathbf{r}(\mathbf{r}\cdot\mathbf{S})}{|\mathbf{r}|^2}\right], \tag{2}$$

where the μ_0 is the vacuum magnetic permeability, g_{Gd}=2 is the g-factor of the electron spin of Gd cluster (S=7/2) and S is its spin vector, μ_B is the Bohr magneton. By positioning the Gd-DO3A complex at the top of the carbon chain with the distance of $\sim$ 22 Å [see Fig. 3(a) in the main text], we observe almost 2 orders of magnitude reduction in T_1, from 0.11 ms to 7.92 μs [see Fig. 4(c) in the main text]. The result is comparable to that of NV centre [49].

SUPPLEMENTARY NOTE 6: ELECTRON SPIN COHERENCE TIME

The central electron spin decoherence in an applied magnetic field $\mathbf{B}$ is caused by the magnetic fluctuations from a large number of nuclear spins, which is described by the following Hamiltonian,

$$H = H_{\mathrm{es}} + H_{\mathrm{bath}} + H_{\mathrm{int}}, \tag{3}$$

where the electron spin Hamiltonian H_{es} is

$$H_{\mathrm{es}} = -\gamma_e\mathbf{B}\cdot\mathbf{S} + DS_Z^2 \tag{4}$$

with the electron gyromagnetic ratio $\gamma_e = -1.76 \times 10^{11}$ rad/(s $\cdot$ T), and D is the zero-field splitting (ZFS). For divacancy in 4H-SiC, the experimental result of $D = 1.336$ GHz, is large enough to prevent electron spin flipping due to the weak hyperfine coupling. Here, we assume the magnetic field direction is along the c-axis of the 4H-SiC, which is defined as the z direction.

The Hamiltonian of the bath nuclear spins is

$$H_{\mathrm{bath}} = -\sum_{(i,\xi)}\gamma_\xi B I_Z^{(i,\xi)} + H_{\mathrm{dip}}, \tag{5}$$

where the composite index (i,ξ) denotes the ith unclear spin of the type ξ. Nuclear spins are coupled by magnetic dipole-dipole interaction of the form

$$H_{\mathrm{dip}} = \frac{1}{2}\sum_{(i,\xi)\neq(i,\xi')}\mathbf{I}^{(i,\xi)}\cdot \mathrm{D}_{i\xi,j\xi'}\cdot\mathbf{I}^{(i,\xi')} = \frac{1}{2}\sum_{(i,\xi)\neq(i,\xi')}\mathbf{I}^{(i,\xi)}\cdot\frac{\mu_0\gamma_\xi\gamma_{\xi'}}{4\pi_{ij}^3}(1-\frac{3\mathbf{r}_{ij}\mathbf{r}_{ij}}{r_{ij}^2})\cdot\mathbf{I}^{(i,\xi')}. \tag{6}$$

$\mathrm{D}_{i\xi,j\xi'}$ is the dipolar coupling tensor between two nuclei located at $\mathbf{r}_{(i,\xi)}$ and $\mathbf{r}_{(i,\xi')}$, and μ_0 is the vacuum permeability.

The defect electron spin couples to the nuclear spins through the hyperfine interaction of the form

$$H_{\mathrm{int}} = \mathbf{S}\cdot \mathrm{A}_{(i,\xi)}\cdot\mathbf{I}^{(i,\xi)}, \tag{7}$$

with the coupling tensor

$$\mathrm{A}_{(i,\xi)} = \frac{\mu_0}{4\pi}\frac{\gamma_e\gamma_\xi}{r_{(i,\xi)}^3}\left[1 - \frac{3\mathbf{r}_{(i,\xi)}\mathbf{r}_{(i,\xi)}}{r_{(i,\xi)}^2}\right]. \tag{8}$$

Since the electron wave function of divacancy is quite localized, we only calculated the hyperfine interaction of the atoms in a small unit cell (SiC: 12.38 Å× 10.72 Å× 20.26 Å, interface: 18.43 Å× 15.96 Å× 65.00 Å). For the atoms

outside of this unit cell, we assume the hyperfine coupling is in dipolar form and calculated it using Eq. (8). Since the hyperfine coupling is much smaller than the ZFS, as long as the system is far from the level-crossing point, we can neglect the electron spin-flipping process.

In free-induction decay (FID) experiments, the coupling to the host nuclear spin induces fast oscillations on the decoherence envelope induced by the bath spin. The coherence decays in several μs. During such a short time, the dipolar interaction between nuclear spins is too weak to take effect. Thus, the FID is caused by the noninteracting nuclear spins, and coherence is the product of the contributions of each single spin,

$$L_{\rm FID}(t) = \prod_j L_{j.\rm FID}(t). \tag{9}$$

For every single spin, the contribution to the FID is

$$L_{j.\rm FID}(t) = \cos\frac{\theta_j^{(0)}}{2}\cos\frac{\theta_j^{(+1)}}{2} + \sin\frac{\theta_j^{(0)}}{2}\sin\frac{\theta_j^{(+1)}}{2}\cos\varphi_j, \tag{10}$$

where $\theta_j^{(m)} = \gamma_n h_j^{(m)}\tau$ and φ_j is the angle between effective fields $\mathbf{h}_j^{(0)}$ and $\mathbf{h}_j^{(+1)}$. The effective fields $\mathbf{h}_j^{(m)}$ is the jth single nuclear spin in a magnetic field $\mathbf{B}$ and a hyperfine field $\mathbf{A}_j^{(m)}$ conditioned on the electron spin state $|m\rangle$. In the weak field, the precession angle about the external field $\theta_j^{(0)} \approx 0$. Therefore, the coherence is

$$L_{\rm FID}(t) = \prod_j \cos\frac{\mathrm{A}_j^{(+1)}t}{2} \approx e^{-\Gamma^2 t^2/2}, \tag{11}$$

where $\mathrm{A}_j^{(+1)}$ is the magnitude of the hyperfine field (Supplementary Table II) of the jth nuclear spin with electron spin in $|+1\rangle$ state and $\Gamma^2 = \sum_j (\mathrm{A}_j^{(+1)})^2/4$ is the FID decay rate in zero fields.

We calculated the electron spin dephasing time for isotope-engineered bulk SiC with ^{29}Si and ^{13}C abundances with $I = 1/2$ nuclear spins at about 0.15% and 0.02%, respectively [24]. The calculated $T_2^* = 48.27$ μs well agrees with the experimentally observed one at $T_2^* = 48.47$ μs (see Ref. 24). Thus we assume that our FID T_2^* values are also accurate for the other cases. For the shallow PL1 beneath deuterium-based (but with natural abundance of ^{13}C spins) alkene-terminated SiC surface (1.3-nm depth) we obtain $T_2^* = 38$ μs which is still long enough to start quantum NMR measurements.

SUPPLEMENTARY NOTE 7: QUANTUM SENSING

Due to the minute energy splitting of nuclear spins under a magnetic field, achieving a sufficiently low entropy state to manifest quantum properties is highly challenging. To address this issue, the electron spin of divacancy is used to transfer polarization to the nuclear spins [51]. The divacancy electron spin can be effectively polarized even at room temperature [16, 52], offering a promising avenue for overcoming the thermalization barrier. In this case, by substituting methyl groups with fluorine groups at the termini of the carbon chain interface [see Fig. 2(a) in the main text], the system incorporates both the electron spin of the divacancy and an array of nuclear spins on the surface. This configuration is governed by the Hamiltonian [51]

$$H = H_{\rm divacancy} + H_{\rm F} + H_{\rm divacancy\text{-}F}. \tag{12}$$

Supplementary Table II. Calculated hyperfine constants in MHz unit for the divacancy defects in SiC bulk, alkene-terminated 4H-SiC when it is perfect, a single methyl group is replaced or all the methyl-groups are replaced by $-CF_3$ groups, and perfluoroheptane (C_7F_{16}) terminated 4H-SiC. The core polarization contribution to the Fermi-contact term is included. C_{n1}-C_{n3}, Si_{n1}-Si_{n3} are the first nearest neighbour atoms, C_{nn1}-C_{nn6} and Si_{nn1}-Si_{nn6} are the second nearest neighbour atoms.

	SiC bulk			Perfect interface			Single $-CF_3$			Layer $-CF_3$			C_7F_{16}		
	A_{xx}	A_{yy}	A_{zz}	A_{xx}	A_{yy}	A_{zz}	A_{xx}	A_{yy}	A_{zz}	A_{xx}	A_{yy}	A_{zz}	A_{xx}	A_{yy}	A_{zz}
C_{n1}	47.09	46.45	118.30	51.30	50.55	122.30	51.23	50.47	122.30	51.37	50.61	122.39	51.40	50.65	122.42
C_{n2}	47.08	46.44	118.29	51.23	50.47	122.30	51.26	50.50	122.21	51.38	50.62	122.35	51.41	50.66	122.42
C_{n3}	47.08	46.44	118.29	51.09	50.33	122.14	51.15	50.39	122.15	51.23	50.47	122.24	51.26	50.50	122.27
Si_{n1}	0.53	-0.44	0.57	0.51	-0.43	0.55	0.50	-0.45	0.53	-0.47	0.48	0.52	0.50	-0.42	0.53
Si_{n2}	0.53	-0.44	0.57	0.56	-0.38	0.59	0.54	-0.40	0.57	0.52	-0.43	0.55	0.53	-0.39	0.56
Si_{n3}	0.53	-0.44	0.57	0.55	-0.39	0.58	0.50	-0.44	0.53	-0.47	0.48	0.52	0.50	-0.42	0.53
C_{nn1}	4.90	4.83	9.95	4.97	4.87	10.12	4.97	4.88	10.13	4.95	4.86	10.10	4.88	4.79	10.01
C_{nn2}	4.90	4.83	9.95	4.95	4.85	10.10	4.96	4.87	10.13	4.94	4.85	10.09	4.87	4.78	10.00
C_{nn3}	4.90	4.83	9.95	4.95	4.85	10.10	4.96	4.86	10.11	4.94	4.84	10.08	4.87	4.78	10.00
C_{nn4}	4.90	4.83	9.95	4.94	4.84	10.10	4.94	4.85	10.10	4.93	4.83	10.07	4.86	4.71	9.98
C_{nn5}	4.90	4.83	9.95	4.94	4.85	10.09	4.95	4.85	10.10	4.93	4.83	10.07	4.86	4.71	9.98
C_{nn6}	4.90	4.83	9.95	4.91	4.81	10.05	4.92	4.83	10.07	4.89	4.80	10.03	4.83	4.75	9.95
Si_{nn1}	-0.36	-0.32	-0.73	-0.35	-0.30	-0.70	-0.35	-0.30	-0.70	-0.36	-0.31	-0.71	-0.35	-0.30	-0.70
Si_{nn2}	-0.36	-0.32	-0.73	-0.35	-0.30	-0.70	-0.35	-0.31	-0.71	-0.36	-0.31	-0.72	-0.35	-0.30	-0.71
Si_{nn3}	-0.36	-0.32	-0.73	-0.35	-0.30	-0.70	-0.35	-0.31	-0.71	-0.36	-0.31	-0.72	-0.35	-0.30	-0.71
Si_{nn4}	-0.36	-0.32	-0.73	-0.35	-0.30	-0.70	-0.35	-0.31	-0.71	-0.36	-0.31	-0.72	-0.35	-0.30	-0.71
Si_{nn5}	-0.36	-0.32	-0.73	-0.34	-0.29	-0.69	-0.35	-0.30	-0.69	-0.35	-0.31	-0.70	-0.34	-0.29	-0.70
Si_{nn6}	-0.36	-0.32	-0.73	-0.34	-0.30	-0.69	-0.35	-0.30	-0.70	-0.35	-0.31	-0.70	-0.34	-0.30	-0.70

The Hamiltonian comprises three terms: the electron spin states of the divacancy, the nuclear spins of the fluorine atoms on the surface, and the interaction between the divacancy electron spin and the nuclear spins (details can be found in Ref. 51). The hyperfine constants of divacancy and F atoms are listed in Supplementary Tables II and III.

For the measurement of the nuclear spin state, the first step involves initialization, where the electron spin of the divacancy is manipulated by a microwave field resonant with the electronic transition between $m_s = 0$ and $m_s = -1$ [53] to achieve the goals of initialization of the quantum simulator, while at the same time decoupling the electron spin of the divacancy from other spin species [54]. The second step is the polarization process. Due to the large zero-field splitting of the divacancy electron spin (1.336 GHz in Ref. 53), direct polarization transfer to nuclear spins is impractical due to significant energy mismatch. To circumvent this, continuous driving of the divacancy electron spin induces microwave-dressed states whose polarization can be efficiently transferred to the nuclear spins when the Hartmann-Hahn condition is satisfied (i.e. when the driving Rabi frequency matches the Larmor frequency of the nuclei). The polarization process involves repetitive cycles where the $\pi/2$ microwave pulse. Efficient polarization transfer occurs when the effective spin-1/2 is resonant with the nuclear spins, achieving dynamical nuclear polarization. The third step is measurement. To measure observables such as the nuclear spin state's structure factors, a gradient

Supplementary Table III. Calculated hyperfine constants in MHz unit for F atoms in alkene-terminated 4H-SiC when a single methyl group is replaced or all the methyl-groups are replaced by $-CF_3$ groups. C-n means the top C atoms of the carbon chains. The core polarization contribution to the Fermi-contact term is included. The distance in the Å unit between the F atom and divacancy is shown after the hyperfine constants.

		F-1				F-2				F-3			
		A_{xx}	A_{yy}	A_{zz}	Distance	A_{xx}	A_{yy}	A_{zz}	Distance	A_{xx}	A_{yy}	A_{zz}	Distance
Single $-CF_3$	C-1	-0.013	-0.012	0.031	26.60	-0.018	-0.014	0.032	26.23	-0.014	-0.008	0.034	26.42
Layer $-CF_3$	C-1	-0.002	-0.004	-0.002	25.81	-0.002	-0.004	-0.003	26.24	-0.004	-0.008	-0.004	26.49
	C-2	-0.002	-0.004	-0.002	25.83	-0.002	-0.004	-0.002	25.90	-0.003	-0.006	-0.003	26.29
	C-3	0.008	0.014	0.007	26.21	0.006	0.010	0.005	26.42	0.003	0.006	0.003	26.59
	C-4	0.006	0.012	0.006	26.12	0.007	0.012	0.006	26.20	0.010	0.016	0.007	26.24
	C-5	-0.005	-0.010	-0.006	26.82	-0.005	-0.010	-0.006	27.05	-0.006	-0.012	-0.006	27.46
	C-6	-0.002	-0.004	-0.002	26.83	-0.006	-0.012	-0.006	27.14	-0.006	-0.012	-0.006	27.50
	C-7	0.001	0.000	-0.001	27.10	-0.008	-0.016	-0.008	27.53	-0.004	-0.008	-0.005	27.82
	C-8	0.002	0.004	0.002	27.55	0.001	0.002	0.001	27.42	0.001	0.002	0.001	27.07
	C-9	0.000	0.000	-0.001	27.29	-0.001	-0.002	-0.001	27.63	0.001	0.000	-0.001	27.76

field is applied to the nuclear spins. This induces position-dependent phases across the nuclear spins due to differing field strengths. The accuracy of these measurements depends on the variation in couplings between the divacancy and individual nuclear spins. In systems with translational invariance, measurements from the divacancy provide information about the average properties of the system state.

SUPPLEMENTARY NOTE 8: MAGNETIC FIELD SENSITIVITY

Shallow PL1 centres are promising candidates for high-precision sensing applications [26]. Positioned just a few nanometers below the surface, these defects provide atomic-scale resolution and substantial sensitivity. The sensitivity η of single PL1 centre is connected to the minimum detectable magnetic field $\delta B_{\min}$ through the relation [28]

$$\eta = \delta B_{\min}\sqrt{\Delta t} \approx \mathcal{P}_{\mathcal{F}} \frac{h}{g\mu_B} \frac{\Delta v}{\mathcal{C}\sqrt{\mathcal{R}}}, \tag{13}$$

where $\mathcal{P}_{\mathcal{F}}$ is a numerical parameter related to the specific profile $\mathcal{F}$ of the spin resonance. For a Gaussian profile, $\mathcal{P}_G = \sqrt{e/8\ln 2} \approx 0.70$, whereas a Lorentzian profile leads to $\mathcal{P}_L = 4/3\sqrt{3} \approx 0.77$. $h \approx 6.626 \times 10^{-34}$ J · s is the Planck constant. $g \approx 2$ is the Landé factor. $\mu_B \approx 9.274 \times 10^{-24}$ J · T^{-1} is the Bohr magneton. $\mathcal{C}$ is the ESR contrast associated with the dip of the PL intensity. $\mathcal{R}$ is the rate of detected photons. Δv is the ESR linewidth, which is fundamentally limited by the inhomogeneous dephasing rate Γ_2^*, which is determined by magnetic dipolar interactions with a bath of spin impurities inside the SiC matrix. These impurities are essentially the nuclear spins associated with isotopes, ^{29}Si and ^{13}C. The effect of the nuclear spin bath can be interpreted as a randomly fluctuating magnetic field applied to the central single spin. In the limit of a large number of bath spins, the distribution of this effective magnetic field is determined by the central limit theorem to be Gaussian [29]. In addition, the ESR linewidth is also

affected by power broadening, both from the continuous laser light used for spin polarization and from the resonant microwave field used for spin rotation. This results in a power-broadened Lorentzian profile of the ESR linewidth. The sharpened ESR linewidth indicates enhanced magnetic field sensitivity. The power broadening can be fully eliminated in ESR spectra by performing pulsed ESR under dark conditions.

For the pulsed-ESR spectroscopy, the ESR linewidth Δv is given by the Fourier transform of the product of the π-pulse rectangular-shaped profile of the duration T_π by the inhomogeneous Gaussian profile, characterized by the coherence time T_2^*. When $T_\pi = T_2^*$, the power broadening is fully cancelled and the ESR shape can be well fitted by a Gaussian profile. In this situation, the rate of the detected photons can be approximated by $\mathcal{R} \approx \mathcal{R}_0 T_L / T_2^*$, where the $\mathcal{R}_0$ is the rate of detected photons for continuous laser excitation. Since $\Delta v = \Gamma_2^* = \frac{2\sqrt{ln2}}{\pi T_2^*}$, the magnetic field sensitivity can then be written as

$$\eta \approx \sqrt{2e}\frac{\hbar}{g\mu_B}\frac{1}{\mathcal{C}\sqrt{\mathcal{R}_0 T_L}}\frac{1}{\sqrt{T_2^*}}. \quad (14)$$

SUPPLEMENTARY NOTE 9: EXPERIMENTAL SETUPS

Here we describe the experimental setups that we used to verify our simulation data reported in the main text and Supplementary Notes. The position of the room-temperature divacancy centres are found by the confocal scanning microscope. The sample was positioned by a piezo-stage (PI, P-611.3S) under a fixed objective lens with an NA of 0.85 (Olympus, LCPLN100XIR). The excitation laser at 914 nm (CNI laser, MXL-H-914) is first modulated by an acoustic optical modulator (GOOCH & HOUSEGO, AOMO 3200-1113), then is filtered by a low-pass filter (Thorlabs, FESH0950) and directed to the objective through the input light path. The input and output path are separated by a dichroic mirror (Semrock, Di02-R980), and the collected emission is driven to the superconductor nanowire single photon detector (Photon Technology Co., SNSPD) through the output path filtered by a long-pass filter (Thorlabs, FELH1000), in order to isolate the excitation laser and observe the emitted photons with wave-lengths longer than 1 μm.

For spin control, a gold wire was drawn to the 4H-SiC surface to deliver the microwaves where the ends of the wire are connected to a microwave source and amplifier (MiniCircuits, SSG6000-RC and MiniCircuits, ZHL-30W-262+) (see Supplementary Fig. 9). Before amplifying, the microwave is modulated by a microwave switch (MiniCircuits, ZASW-2-50DRA+) in order to produce different control sequences. The total measurement sequences and timing synchronizations are controlled by a pulse blaster board (SpinCore, PulseBlasterESR-PRO), and the data is acquired by a data acquisition card (National Instrument, USB-6341).

SUPPLEMENTARY NOTE 10: CONFOCAL SCANNING IMAGES FOR SIC SAMPLES WITH DIVACANCIES.

In naturally abundant SiC, three samples, Sample I (SI), Sample II (SII) and Sample III (SIII), were prepared to investigate the properties of single PL1 and PL6 divacancies. The details of the Samples can be found in the

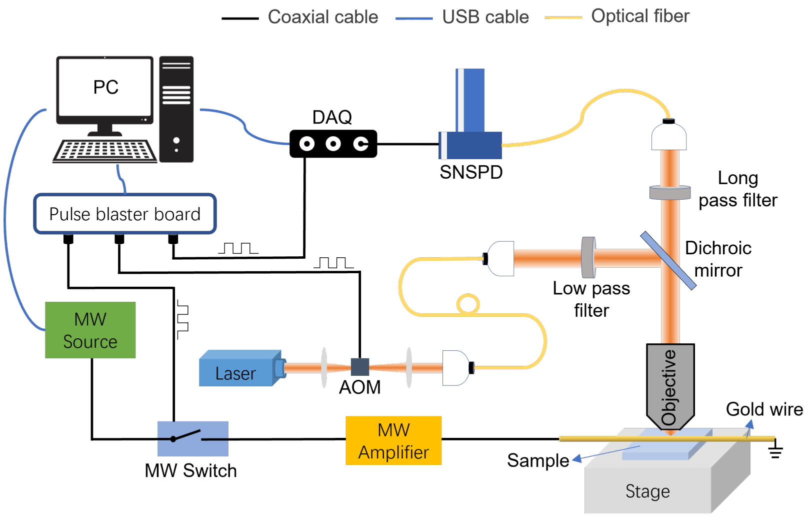

Supplementary Fig. 9. Schematic figures about the sample with antenna and the optical setup [55]. A 914-nm laser is modulated by an acousto-optic modulator (AOM), then focused onto the sample by an objective after reflection by a dichroic mirror (DM). Fluorescence emitted from the sample is collected by the same objective, filtered through an interference filter (IF), and detected by a superconducting single photon detector (SSPD). A Ω shape antenna on the sample generates microwave (MW) pulses. Electrical pulse sequences for the AOM and MW systems are controlled by a personal computer (PC).

Methods section of the main text. All the samples were implanted with carbon ions at a dose of 1×10^{11} ions/cm^2 and annealed at 900 °C degrees for half an hour to create single divacancy defects. The only difference between the samples is the implanting energy, 1 keV for SI and SII and 5 keV for SIII, resulting in the central implantation depths of 2 nm and 7 nm, respectively, which was calculated by the Stopping and Range of Ions in Matter (SRIM) method (see Supplementary Fig. 10).

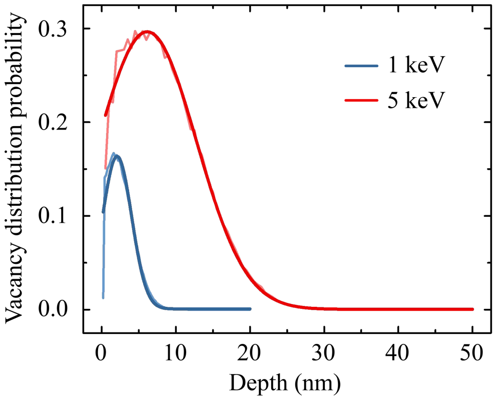

Supplementary Fig. 10. Simulated probability distribution of divacancy generation in SiC as a function of depth during carbon ion implantation with the energy of 1 keV (blue) and 5 keV (red).

After the implantation and annealing process, the surfaces are typically treated with HF and piranha solution to remove the defective oxide layer. To further characterize the chemical composition of the treated surface, X-ray photoelectron spectroscopy (XPS) was performed on SI and SII. The results, shown in Supplementary Fig. 11, reveal

that after the treatment, fluorine content remains minimal (below 1%), while oxygen is still present as a significant component. This indicates that rather than forming a fluorinated surface, the dominant surface termination consists of hydroxyl (−OH) groups, which are likely to result from the reaction between the solution and native oxide layer. The result is consistent with previous experimental reports [42, 43]. Additionally, the presence of residual oxygen suggests that some oxide-related defects may still remain after the treatment.

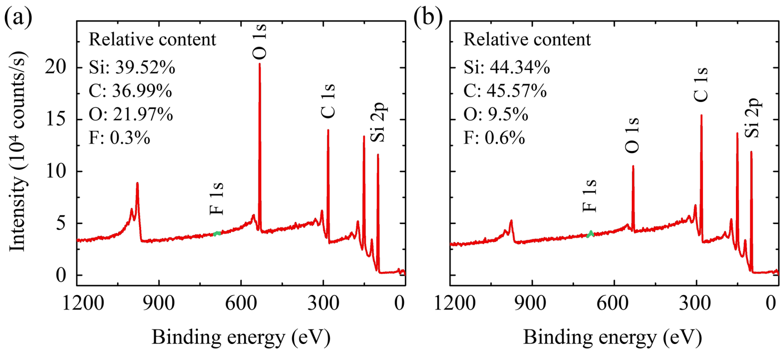

Supplementary Fig. 11. X-ray photoelectron spectroscopy (XPS) spectra of (a) SI and (b) SII after piranha and HF solution treatment.

Following the surface characterization, confocal fluorescence imaging was conducted to investigate the impact of HF treatment on defect stability. Supplementary Figs. 12(a) and (b) show confocal images of two individual PL1 defects in SI, labeled PL1-P1-SI and PL1-P2-SI, highlighted with red circles. PL1-P1-SI was measured after annealing, while PL1-P2-SI was measured after 30 minutes of HF immersion. The image in Supplementary Fig. 12(b) appears visibly cleaner than that in Supplementary Fig. 12(a) due to the HF treatment, suggesting that HF removed the defective oxide layer. As a result, PL1-P1-SI was no longer detectable. Similarly, Supplementary Figs. 12(c) and (d) show confocal images of two single PL6 defects in SII, labeled PL6-P1-SII and PL6-P2-SII, also marked with red circles. The surface of SII after HF immersion exhibits comparable characteristics to those observed in SI, further corroborating the cleaning effect of the HF treatment.

Moreover, deeper divacancy defects in SIII demonstrate similar trends, reinforcing these findings. To further investigate the impact of surface treatment on defect coherence, reactive ion etching (RIE) was employed in SIII to remove 9 nm of material using oxygen (O_2) and sulfur hexafluoride (SF_6). Comparing with the confocal image before etching shown in Supplementary Fig. 13(a), the image after etching presented in Supplementary Fig. 13(b) reveals the detection of only one single PL6 defect. Given that the etching depth (9 nm) surpasses the central implantation depth (7 nm) of SIII, this suggests that the removal of the surface layer significantly affects the density and spatial distribution of detectable defects. The results further corroborate that O_2 and SF_6 etching forms fluorine/oxygen-containing surface compounds, leading to an effect similar to that observed with HF treatment.

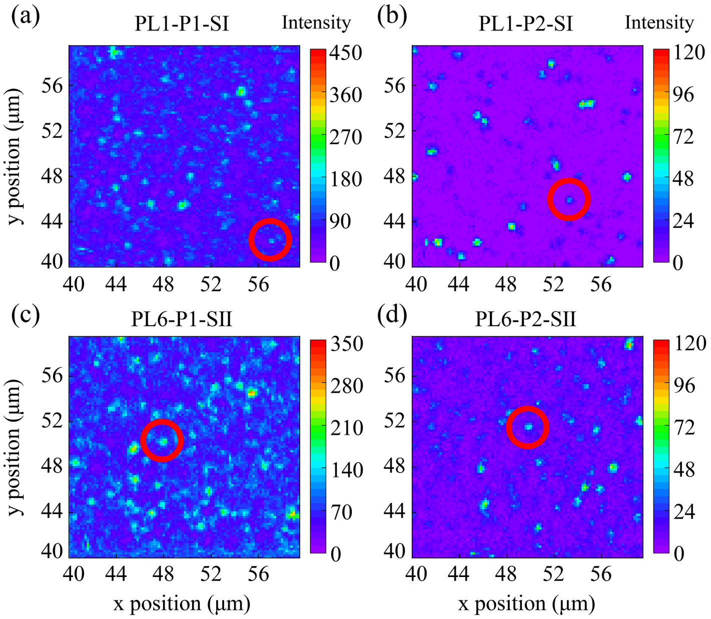

Supplementary Fig. 12. Confocal scanning images of PL1 and PL6 defects in SI and SII. Confocal scanning images of (a) PL1-P1-SI after annealing and (b) PL1-P2-SI after HF immersion for 30 minutes are highlighted with red circles. (c) and (d) Confocal scanning images of PL6-P1-SII and PL6-P2-SII in SII after annealing and HF immersion.

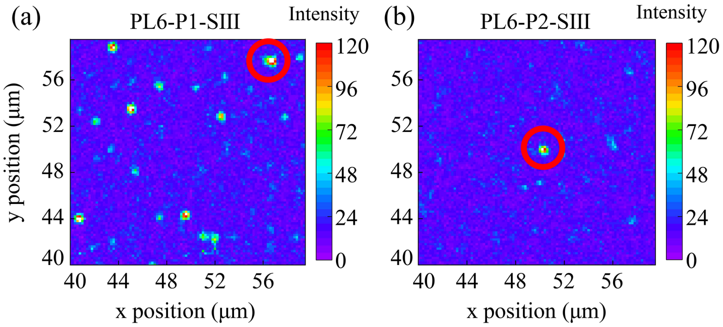

Supplementary Fig. 13. Confocal scanning image of PL6 defects in SIII (a) before and (b) after reactive ion etching using oxygen (O_2) and sulfur hexafluoride (SF_6).

SUPPLEMENTARY NOTE 11: ODMR AND RABI OSCILLATION

To characterize the defects, we performed both continuous-wave ODMR (cw-ODMR) and Rabi oscillation measurements to determine the resonant frequency and the corresponding π pulse. Supplementary Fig. 14 presents the cw-ODMR and Rabi oscillation results for the PL1 (PL1-P2-SI) and PL6 (PL6-P2-SII) defects at room temperature, measured in a magnetic field of 180 G applied parallel to the c-axis. The cw-ODMR spectra were fitted using a Lorentzian function to extract the centre frequency of the dip, while the Rabi oscillations were fitted with an exponentially decaying cosine function to determine the oscillation period.

As the magnetic field applied is relatively large (180 G), the separation between the cw-ODMR peaks becomes

approximately 1 GHz. Thus, the cw-ODMR spectra in Supplementary Figs. 14(a) and (c) only show the left portion of the spectrum. By utilizing the divacancy g-factor of 2.8 MHz/G, the zero-field position of the cw-ODMR peak can be inferred. For instance, the observed peak at 802 MHz [see Supplementary Fig. 14(a)] for PL1 can be shifted by 180 G $\times$ 2.8 MHz/G = 504 MHz, yielding a calculated zero-field frequency of approximately 1.306 GHz, which agrees well with previously reported experimental values (1.317 MHz in Ref. 26). This confirms that the defect identified as PL1 is consistent with the expected characteristics. Similarly, the experimental results (1.356 MHz) for PL6 in Supplementary Fig. 14(c) also match prior findings (1.351 MHz in Ref. 26), validating the authenticity of the measured defects.

The comparison of cw-ODMR spectra and Rabi oscillations for both PL1 and PL6 reveals a marked difference in contrast, with PL1 exhibiting significantly lower contrast (approximately 1.2%) compared to PL6 (approximately 12%). As a result, a higher number of data accumulation cycles were required to obtain accurate measurements of the Ramsey oscillations and determine the T_2^* values for PL1.

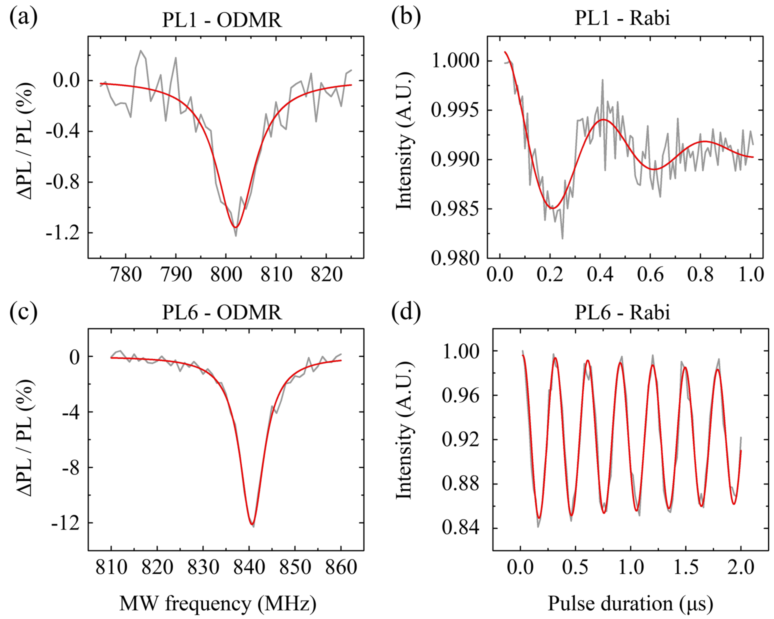

Supplementary Fig. 14. The cw-ODMR spectra and Rabi oscillation results for PL1 (PL1-P2-SI) and PL6 (PL6-P2-SII) defects measured in a magnetic field of 180 G. (a) and (c) show the cw-ODMR results for PL1 and PL6, respectively. (b) and (d) show the Rabi oscillations for PL1 and PL6, respectively. The black lines are the experimental raw data and the red lines are the corresponding theoretical fittings. The cw-ODMR dips were fitted using Lorentz function, $y = y_0 + (2A/\pi) \times (w/4(x - x_c)^2 + w^2)$, where y_0, A, and w are free parameters, x_c is the centre frequency of the dip. The Rabi oscillations were fitted using $y = a + b \times e^{-x/c} \times \cos(2\pi x/T + d)$, where a, b, c and d are free parameters, T is the period of the oscillation.

SUPPLEMENTARY NOTE 12: SPIN-LATTICE RELAXATION TIME: T_1

The relaxometry sensing – based on the observation of the T_1 time of the quantum sensor – is one of the most successful applications of quantum sensors in bio-sensing [45]. To measure the T_1 time of divacancy, we performed

a standard inversion-recovery pulse sequence, in which a π pulse inverts the spin population, followed by a variable delay before a readout pulse detects the spin state. The relaxation time T_1 is then extracted by fitting the decay of the photoluminescence signal.

We investigated the T_1 time of shallow PL6 centres in SII (named PL6-P3-SII and PL6-P4-SII). The corresponding cw-ODMR spectra and Rabi oscillations are shown in Supplementary Fig. 15. The cw-ODMR read-out contrasts were measured to be 8.1% for PL6-P3-SII and 8.8% for PL6-P4-SII, with the surface covered by an oxide layer. The surface treatment process is illustrated in Supplementary Fig. 16. First, the sample was cleaned in a piranha solution at 90°C in a water bath for 2 hours, followed by immersion in GR-grade HF solution for 30 minutes to remove the insulating amorphous SiO_2 (a-SiO_2) layer. After rinsing with deionized water to remove residual HF, the surface was immediately activated by sonication in alcohol. Subsequently, 2.5 μL of 1-alkene was dropped onto the surface and allowed to evaporate naturally. Following this alkene termination, the ODMR contrasts increased to 10.6% for PL6-P3-SII and 9.9% for PL6-P4-SII [see Supplementary Figs. 15(b) and (d)]. This enhancement indicates that alkene termination improves the spin readout contrast. To further investigate surface magnetic noise effects, 2.5 μL Gd-DO3A solution was dropped onto the surface and allowed to evaporate naturally as well.

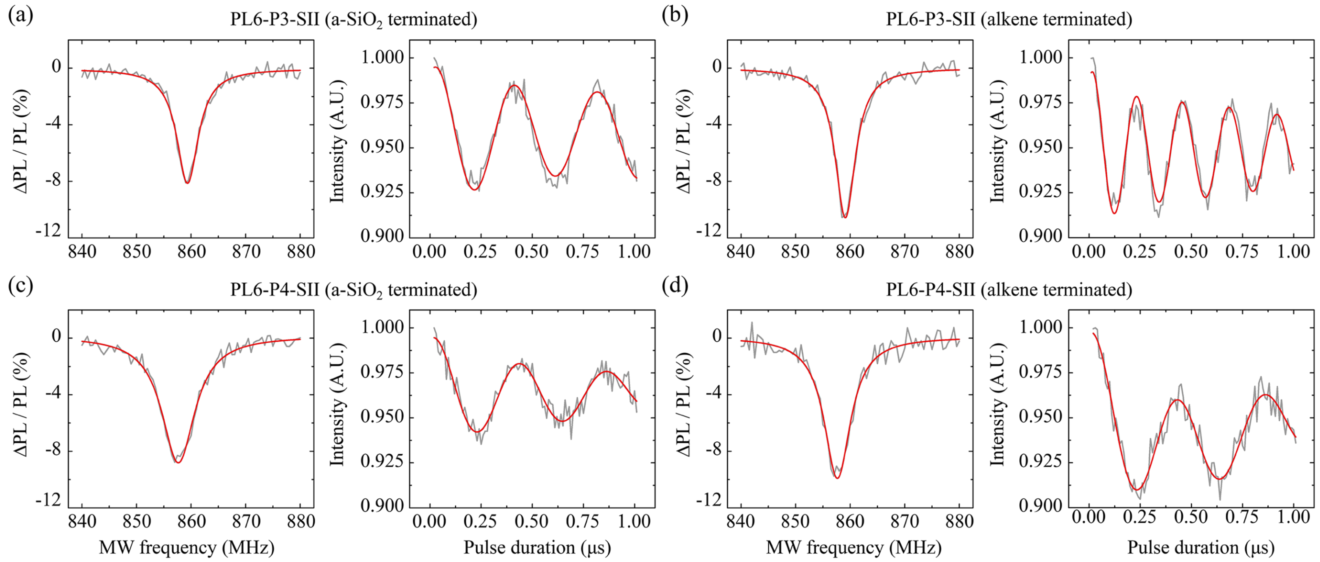

Supplementary Fig. 15. The cw-ODMR spectra and Rabi oscillation results for PL6 (PL6-P3-SII and PL6-P4-SII) defects in a-SiO_2 and alkene terminated SiC surface.

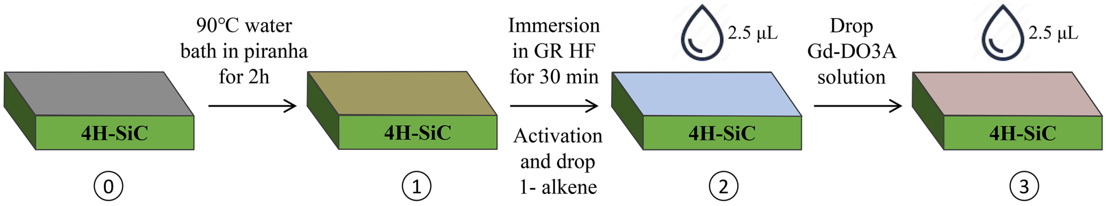

Supplementary Fig. 16. The procedure of SiC surface treatments in the experiment.

According to the XPS result obtained after step 3 [see Supplementary Fig. 17(b)], the layers were successfully formed on the surface since the signal of the Si element almost disappears and the Gd element emerges. The concentration of Gd-DO3A solution was 5 μg/mL, and given the molar mass of Gd-DO3A (499.60 g/mol), the calculated molecular coverage on the 5 mm $\times$ 5 mm sample surface is approximately 1.21 molecules/nm^2, which is consistent with the values used in theoretical calculations. After each treatment step, the corresponding T_1 times of the same PL6 colour centres (labeled by step numbers) were measured, as shown in Supplementary Fig. 18.

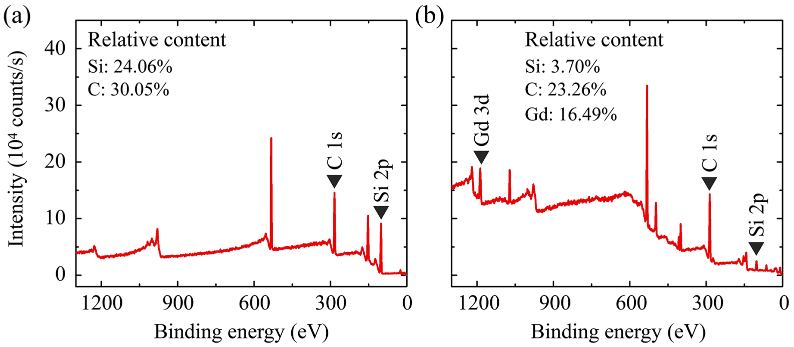

Supplementary Fig. 17. XPS spectra after the treatment of (a) step 2 and (b) step 3.

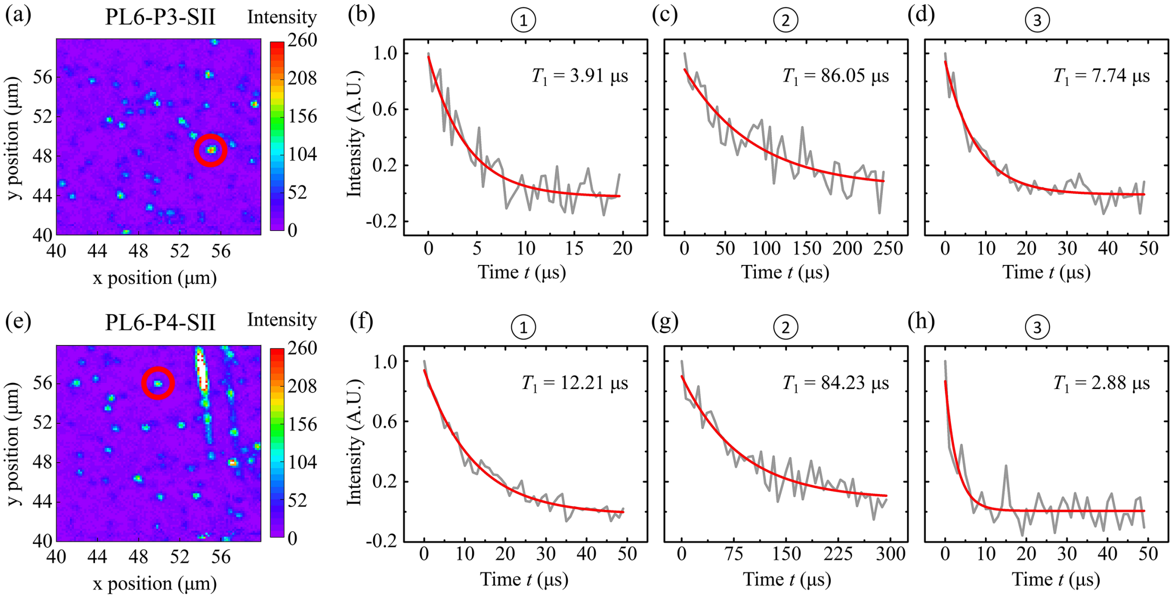

Supplementary Fig. 18. Confocal scanning images and T_1 times of two single near-surface PL6 centres. (a) and (e): Confocal scanning images of PL6-P3-SII and PL6-P4-SII, respectively. (b) to (d): T_1 times of PL6-P3-SII corresponding to different treatment steps. (f) to (h): T_1 times of PL6-P4-SII corresponding to different treatment steps.

Here we measured the T_1 times of the two near-surface single PL6 centres (PL6-P3-SII and PL6-P4-SII). As shown in Supplementary Figs. 18(b) and (f), the T_1 times of these centres with the a-SiO_2 layer present are significantly shorter than those located deeper in the bulk. This highlights the detrimental effect of surface oxidation on the T_1 time. After removing the a-SiO_2 layer and subsequent coating the surface with a layer of 1-alkene in step 2,

the T_1 times shown in Supplementary Figs. 18(c) and (g) increased by approximately an order of magnitude. This improvement is attributed to the 1-alkene layer effectively passivating the surface and suppressing re-oxidation, thus reducing magnetic noise from surface-related defects. In step 3, after applying an additional layer of Gd-DO3A on top of the 1-alkene, the T_1 times decreased again to serval microseconds [see Supplementary Figs. 18(d) and (h)], clearly demonstrating the influence of Gd-induced magnetic fluctuations on the T_1 time of near-surface PL6 centres. These results are consistent with our theoretical predictions in the main text and confirm the sensitivity of shallow divacancy centres to local magnetic noise, which opens a promising avenue for high-sensitivity quantum sensing based on near-surface colour centres in SiC.

To investigate the intrinsic spin properties of divacancies in SiC, we measured T_1 for deep defects created through nitrogen implantation at 60 keV, yielding an estimated defect depth of approximately 100 nm, as confirmed by SRIM simulations. In this deep region, the measured T_1 time is 112.46 μs [see Supplementary Fig. 19(b)]. This value aligns well with previously reported results [16, 26, 56].

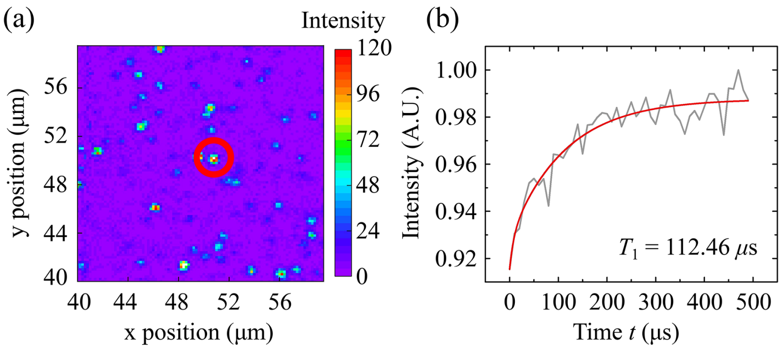

Supplementary Fig. 19. (a) Confocal scanning image of PL6 defects in SII. (b) The measured T_1 time by fitting the decay of the photoluminescence signal.

SUPPLEMENTARY NOTE 13: TRANSVERSE RELAXATION TIME: T_2^*

After characterizing the T_1 relaxation time, we next investigated the T_2^* time to gain insights into the coherence properties of the spin system. To measure the T_2^* time, we employed a Ramsey sequence, as illustrated in Supplementary Fig. 20. Initially, the spin is prepared in the ground state using a laser pulse. A $\pi/2$ microwave pulse is then applied, rotating the spin state into the xy-plane of the Bloch sphere. After this, the spin undergoes free evolution for a variable duration τ, during which phase noise gradually accumulates. A second $\pi/2$ pulse is applied after the evolution time to readout the spin state, revealing a decrease in coherence as τ increases. This decay in coherence, due to phase accumulation during the free evolution, leads to a reduction in the Ramsey fringe contrast. Supplementary Fig. 20(a) shows the schematic of the Ramsey sequence used in our experiments, while Fig. 20(b) depicts the evolution of the spin on the Bloch sphere under microwave pulse manipulation.

The T_2^* times of PL1 in SI (PL1-P2-SI) and PL6 in SII (PL6-P2-SII) are shown in Supplementary Fig. 21 and Fig. 22, respectively. For PL1-P2-SI as illustrated in Supplementary Fig. 21(a), after treatments using HF and

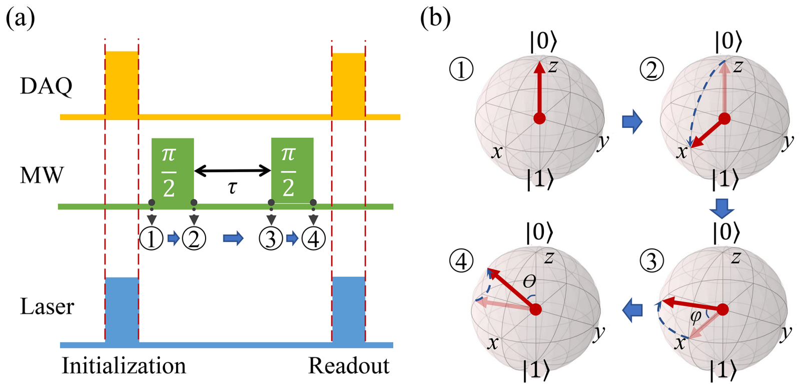

Supplementary Fig. 20. (a) Schematic of the Ramsey sequence used in this work. (b) Evolution of the spin on the Bloch sphere under manipulation by the microwave pulse.

piranha solution, the T_2^* values are 0.42 μs and 0.51 μs, respectively. The T_2^* value is lower than that observed in bulk SiC, likely due to the presence of residual surface functional groups such as $-$OH and $-$F, which form after HF and piranha treatment. Since the divacancy defects are located close to the surface, the coherence time is influenced by the nuclear spins of fluorine and hydrogen atoms, introducing additional magnetic noise and limiting spin coherence.

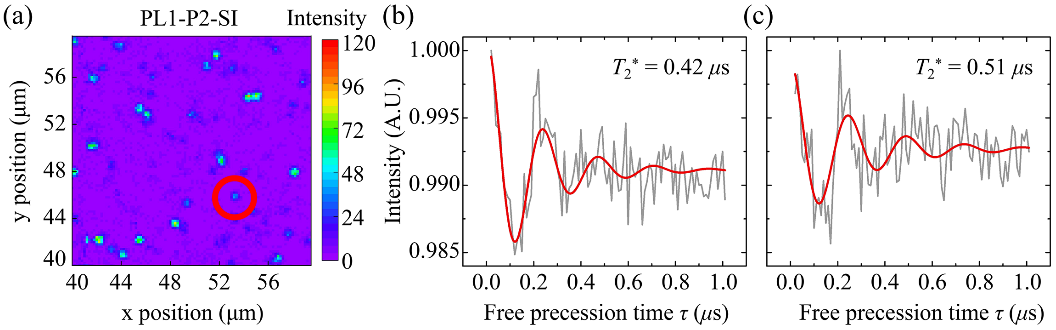

Supplementary Fig. 21. The experimental results of PL1 in SI. (a) Confocal scanning image. (b) and (c) show the Ramsey oscillation after surface treatment with HF and piranha solution, respectively.

A similar trend is observed for PL6-P2-SII (see Supplementary Fig. 22). After HF and piranha treatments, the T_2^* values are 0.98 μs and 1.12 μs, respectively. These results further confirm that surface impurities, defects, and functional groups play a crucial role in determining the coherence time of quantum qubits in SiC surface.

The observed difference in T_2^* between PL6 and PL1 can be attributed to the different sensitivities of the two qubits to spin noise. Compared to PL1, PL6 is expected to be less sensitive to spin noise, which is a dominant decoherence source near the surface. Consequently, under the same nuclear spin environment, PL6 exhibits a longer coherence time in near-surface conditions. This suggests that spin fluctuations, likely caused by residual surface functional groups or defects, play a significant role in determining the coherence properties of shallow divacancy qubits. By combining the currently observed room-temperature optical readout contrast (1.2% for PL1 and 12% for PL6 in Supplementary Note 11), the projected magnetic field sensitivity are ~ 750 nT/$\sqrt{\text{Hz}}$ and ~ 50 nT/$\sqrt{\text{Hz}}$, respectively.

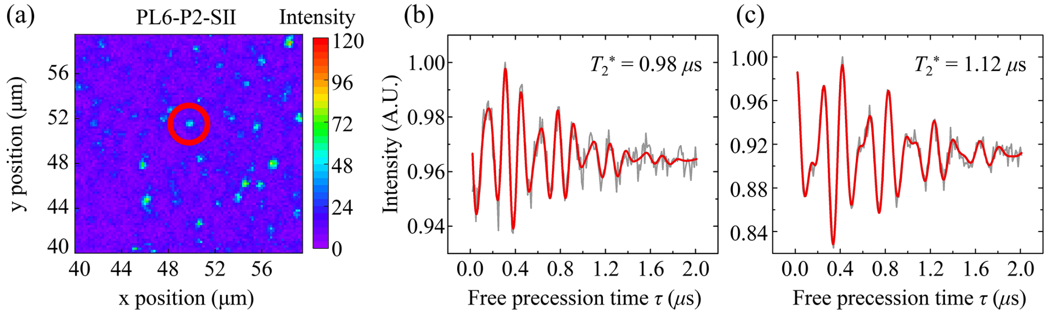

Supplementary Fig. 22. The experimental results of PL6 in SII. (a) Confocal scanning image. (b) and (c) show the Ramsey oscillation after surface treatment with piranha and HF solution, respectively.

To further investigate the influences of surface -F groups on the coherence properties of near-surface divacancy centres, we coated the SII surface with perfluoroheptane (C_7F_{16}) and measured the T_2^* times of the PL6-P3-SII centre, as shown in Supplementary Fig. 23. The coating procedure followed the same protocol outlined in Supplementary Fig. 16. As a reference, the T_2^* time of the PL6 in the naturally abundant SiC surface functionalised with alkene(H) was 1 μs [see Supplementary Fig. 23(a)]. After applying C_7F_{16} solutions with mass ratios of 8.5% and 85%, the T_2^* times showed a slight decrease with increasing fluorine concentration, as presented in Supplementary Figs. 23(b) and (c). Although the concentration of fluorine atoms increased by an order of magnitude, the observed reduction in T_2^* was relatively modest. This is because T_2^* is primarily determined by the quasi-static magnetic noise from all surrounding nuclear spins, not just the surface species. In our system, naturally abundant SiC was used as the host material, in which 1.1% of the carbon atoms and 4.7% of silicon atoms possess an $I = 1/2$ nuclear spin. These nuclear spins form an intrinsic spin bath that contributes significantly to magnetic noise and limits the spin coherence time. In addition to this bulk contribution, hydrogen atoms commonly present on the surface further increase the local magnetic noise through their nuclear spins. Consequently, the effect of fluorine nuclei is superimposed on an already dense and noisy spin environment. As a result, the incremental dephasing caused by fluorine spins is relatively small, leading to only a modest change in the overall T_2^* time. This observation is consistent with our simulation results [see Supplementary Fig. 24(a)], which show that replacing surface hydrogen atoms with fluorine leads to only a slight reduction in T_2^* when the spin bath from bulk nuclear spins is not suppressed via isotopic purification.

To induce a more pronounced impact on the coherence properties of near-surface divacancy centres, we functionalized the SiC surface with Gd-DO3A molecules. Three different concentrations of Gd-DO3A solution were used, corresponding to surface Gd densities of approximately 0.1, 1, and 2 molecules/nm^2, respectively, as detailed in Supplementary Note 12. The corresponding T_2^* times of the same PL6 colour centre were measured after each treatment and are shown in Supplementary Figs. 23(d)–(f). A clear reduction in T_2^* was observed with increasing Gd coverage, especially between the 1 and 2 molecules/nm^2 conditions, indicating that the strong magnetic noise introduced by Gd clusters significantly affects the dephasing dynamics of the spin qubit. This experimental trend is well reproduced by our simulations [see Supplementary Fig. 24(b)], which also show that magnetic fluctuations from high-density Gd spins can drastically reduce the T_2^* time.

To investigate the intrinsic spin properties of divacancies in SiC, we measured T_2^* time for deeper divacancies created

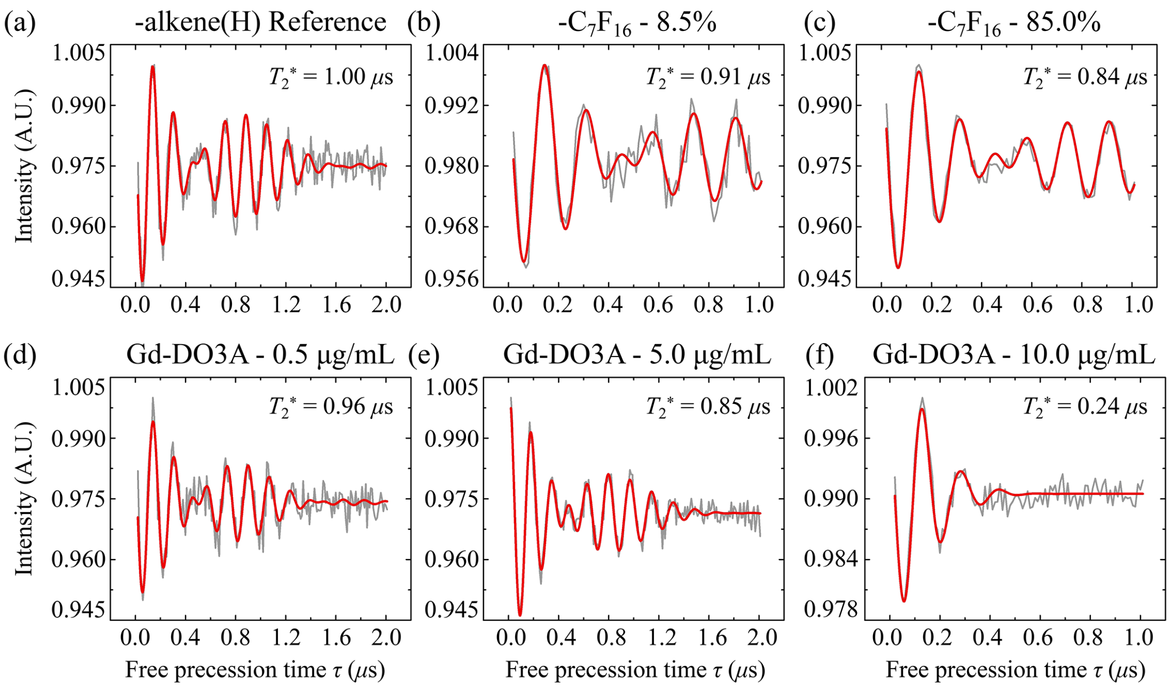

Supplementary Fig. 23. Experimental investigation of the effect of surface F or Gd ions on T_2^* for about ∼ 2-nm deep single divacancy PL6 centre. (a) Ramsey oscillation for the PL6 in the naturally abundant SiC surface functionalised with alkene(H). (b) and (c) show the Ramsey oscillation after surface coating with perfluoroheptane (C_7F_{16}) solutions of different dilution concentrations, respectively. (d) - (f) show Ramsey oscillations after surface coating with Gd-DO3A solutions of different dilution concentrations, respectively.

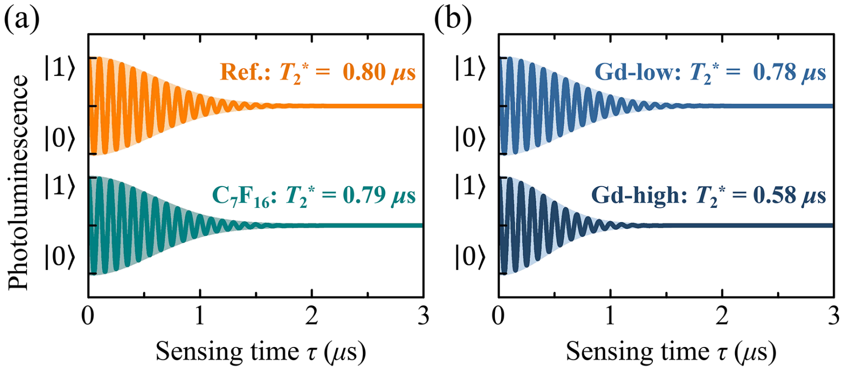

Supplementary Fig. 24. Simulation results of T_2^* times of divacancy in SiC surface functionalized with carbon chain (Ref.), C_7F_{16}, and Gd-DO3A at low (Gd-low) and high (Gd-high) concentrations.

through carbon implantation at 5 keV in SIII, yielding an estimated defect depth of approximately 7 nm as confirmed by SRIM simulations (see Supplementary Note 10). In this deeper region, the measured T_2^* time increases to 1.42 μs (see Supplementary Fig. 25), which is consistent with the previously reported experimental values [16, 57].

The simulation results of T_2^* are summarized in Supplementary Table IV. For the divacancy at the SiC surface with −OH termination, a T_2^* value of 0.80 μs is obtained for the divacancy located 1.3 nm below the surface. For

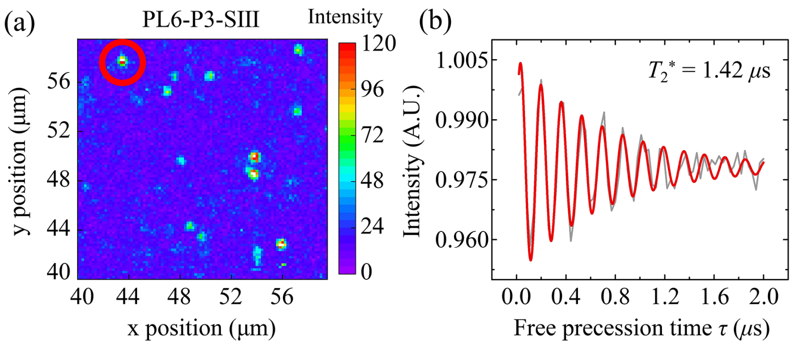

Supplementary Fig. 25. Experimental observation of (a) confocal scanning image and (b) Ramsey oscillation for about $\sim$ 7-nm deep single PL6 centre in the naturally abundant SiC (SIII).

$-$F termination, the T_2^* value is slightly higher at 0.82 μs. However, considering the XPS results presented in Supplementary Fig. 11, the HF-treated surface is not purely terminated with $-$OH groups but also contains a small fraction of fluorine atoms. To better reflect this experimental reality, we further simulated mixed terminations with varying fluorine concentrations, ranging from 2.8% to 16.7%. As shown in Supplementary Table IV, the T_2^* values for these mixed-termination surfaces remain around 0.80 μs, indicating that a small proportion of fluorine has minimal impact on the coherence time of shallow divacancies. Since the simulated T_2^* values exhibit minimal variation with increasing fluorine concentration, we infer that the experimentally observed improvement in T_2^* following surface treatments (such as HF and piranha etching) is likely due to the removal of extrinsic surface defects rather than changes in the intrinsic chemical termination. Furthermore, considering the influence of the nuclear spins of nitrogen atoms in the experimental SiC samples, the simulation results are generally consistent with the measured experimental data.

Supplementary Table IV. T_2^* of divacancy in SiC surface with $-$OH, $-$F and Mix termination.

Naturally abundant SiC	depth (nm)	T_2^* (μs)
$-$OH termination	0.3	0.4799
	0.8	0.6279
	1.3	0.7954
$-$F termination	1.3	0.8198
Mix (2.8% $-$F)	1.3	0.7960
Mix (5.6% $-$F)	1.3	0.7971
Mix (8.3% $-$F)	1.3	0.7972
Mix (16.7% $-$F)	1.3	0.7989

[1] J. M. Knaup, P. Deák, T. Frauenheim, A. Gali, Z. Hajnal, and W. Choyke, Phys. Rev. B **71**, 235321 (2005).

[2] V. V. Afanas'ev, F. Ciobanu, S. Dimitrijev, G. Pensl, and A. Stesmans, in *Materials Science Forum*, Vol. 483 (Trans Tech Publ, 2005) pp. 563–568.

[3] T. Kobayashi and Y.-i. Matsushita, J. Appl. Phys. **126**, 145302 (2019).

[4] F. Devynck, A. Alkauskas, P. Broqvist, and A. Pasquarello, Phys. Rev. B **83**, 195319 (2011).

[5] Z. Zhang, Z. Wang, Y. Guo, and J. Robertson, Appl. Phys. Lett. **118**, 031601 (2021).

[6] P. Li, P. Udvarhelyi, S. Li, B. Huang, and A. Gali, Phys. Rev. B **108**, 085201 (2023).

[7] C. Chatillon and F. Teyssandier, J. Eur. Ceram. Soc. **42**, 1175 (2022).

[8] M. Boero, A. Pasquarello, J. Sarnthein, and R. Car, Phys. Rev. Lett. **78**, 887 (1997).

[9] Y. Kagoyama, M. Okamoto, T. Yamasaki, N. Tajima, J. Nara, T. Ohno, H. Yano, S. Harada, and T. Umeda, J. Appl. Phys. **125**, 065302 (2019).

[10] P. Dong, P. Li, L. Zhang, H. Tan, Z. Hu, K. Zhou, Z. Li, X. Yu, J. Li, and B. Huang, Phys. Rev. Appl. **15**, 034007 (2021).

[11] B. Johnson, J. Woerle, D. Haasmann, C.-K. Lew, R. Parker, H. Knowles, B. Pingault, M. Atature, A. Gali, S. Dimitrijev, M. Camarda, and J. McCallum, Phys. Rev. Appl. **12**, 044024 (2019).

[12] A. Gali, E. Janzén, P. Deák, G. Kresse, and E. Kaxiras, Phys. Rev. Lett. **103**, 186404 (2009).

[13] A. Gali, Nanophotonics **8**, 1907 (2019).

[14] J. P. Perdew, K. Burke, and M. Ernzerhof, Phys. Rev. Lett. **77**, 3865 (1996).

[15] A. Alkauskas, B. B. Buckley, D. D. Awschalom, and C. G. Van de Walle, New J. Phys. **16**, 073026 (2014).

[16] W. F. Koehl, B. B. Buckley, F. J. Heremans, G. Calusine, and D. D. Awschalom, Nature **479**, 84 (2011).

[17] V. Ivády, K. Szasz, A. L. Falk, P. V. Klimov, E. Janzén, I. A. Abrikosov, D. D. Awschalom, and A. Gali, in *Materials Science Forum*, Vol. 858 (Trans Tech Publ, 2016) pp. 322–325.

[18] A. Csore, B. Magnusson, N. T. Son, A. Gällström, T. Ohshima, I. Ivanov, and A. Gali, in *Materials Science Forum*, Vol. 963 (Trans Tech Publ, 2019) pp. 714–717.

[19] D. Beke, J. Valenta, G. Ka´rolyha´zy, S. Lenk, Z. Cziga´ny, B. G. Ma´rkus, K. Kamara´s, F. Simon, and A. Gali, J. Phys. Chem. Lett. **11**, 1675 (2020).

[20] N. T. Son and I. G. Ivanov, J. Appl. Phys. **129** (2021), https://doi.org/10.1063/5.0052131.

[21] Z.-X. He, J.-Y. Zhou, Q. Li, W.-X. Lin, R.-J. Liang, J.-F. Wang, X.-L. Wen, Z.-H. Hao, W. Liu, S. Ren, *et al.*, Nat. Commun. **15**, 10146 (2024).

[22] E. M. Lee, A. Yu, J. J. de Pablo, and G. Galli, Nat. Commun. **12**, 6325 (2021).

[23] R. Nagy, M. Niethammer, M. Widmann, Y.-C. Chen, P. Udvarhelyi, C. Bonato, J. U. Hassan, R. Karhu, I. G. Ivanov, N. T. Son, *et al.*, Nat. Commun. **10**, 1954 (2019).

[24] A. Bourassa, C. P. Anderson, K. C. Miao, M. Onizhuk, H. Ma, A. L. Crook, H. Abe, J. Ul-Hassan, T. Ohshima, N. T. Son, *et al.*, Nat. Mater. **19**, 1319 (2020).

[25] C. Babin, R. Stöhr, N. Morioka, T. Linkewitz, T. Steidl, R. Wörnle, D. Liu, E. Hesselmeier, V. Vorobyov, A. Denisenko, *et al.*, Nat. Mater. **21**, 67 (2022).

[26] Q. Li, J.-F. Wang, F.-F. Yan, J.-Y. Zhou, H.-F. Wang, H. Liu, L.-P. Guo, X. Zhou, A. Gali, Z.-H. Liu, *et al.*, Natl. Sci. Rev. **9**, nwab122 (2022).

[27] M. Widmann, S.-Y. Lee, T. Rendler, N. T. Son, H. Fedder, S. Paik, L.-P. Yang, N. Zhao, S. Yang, I. Booker, *et al.*, Nat. Mater. **14**, 164 (2015).

[28] A. Dréau, M. Lesik, L. Rondin, P. Spinicelli, O. Arcizet, J.-F. Roch, and V. Jacques, Phys. Rev. B **84**, 195204 (2011).

[29] V. Dobrovitski, A. Feiguin, D. Awschalom, and R. Hanson, Phys. Rev. B **77**, 245212 (2008).

[30] J. M. Taylor, P. Cappellaro, L. Childress, L. Jiang, D. Budker, P. Hemmer, A. Yacoby, R. Walsworth, and M. Lukin, Nat. Phys. **4**, 810 (2008).

[31] D. Simin, V. Soltamov, A. Poshakinskiy, A. Anisimov, R. Babunts, D. Tolmachev, E. Mokhov, M. Trupke, S. Tarasenko, A. Sperlich, *et al.*, Phys. Rev. X **6**, 031014 (2016).

[32] M. Niethammer, M. Widmann, S.-Y. Lee, P. Stenberg, O. Kordina, T. Ohshima, N. T. Son, E. Janzén, and J. Wrachtrup, Phys. Rev. Appl. **6**, 034001 (2016).

[33] J. B. Abraham, C. Gutgsell, D. Todorovski, S. Sperling, J. E. Epstein, B. S. Tien-Street, T. M. Sweeney, J. J. Wathen, E. A. Pogue, P. G. Brereton, *et al.*, Phys. Rev. Appl. **15**, 064022 (2021).

[34] I. Lekavicius, S. Carter, D. Pennachio, S. White, J. Hajzus, A. Purdy, D. Gaskill, A. Yeats, and R. Myers-Ward, Phys. Rev. Appl. **19**, 044086 (2023).

[35] K. Tahara, S.-i. Tamura, H. Toyama, J. J. Nakane, K. Kutsuki, Y. Yamazaki, and T. Ohshima, npj Quantum Inf. **11**, 58 (2025).

[36] P. Fisher, A. Zappacosta, J. Fuhrmann, B. Haylock, W. Gao, R. Nagy, F. Jelezko, and R. Cernansky, Nano Lett. (2025), 10.1021/acs.nanolett.5c02515.

[37] A. L. Falk, B. B. Buckley, G. Calusine, W. F. Koehl, V. V. Dobrovitski, A. Politi, C. A. Zorman, P. X.-L. Feng, and D. D. Awschalom, Nat. Commun. **4**, 1819 (2013).

[38] G. Wolfowicz, C. P. Anderson, A. L. Yeats, S. J. Whiteley, J. Niklas, O. G. Poluektov, F. J. Heremans, and D. D. Awschalom, Nat. Commun. **8**, 1876 (2017).

[39] M. Rühl, C. Ott, S. Götzinger, M. Krieger, and H. Weber, Appl. Phys. Lett. **113**, 122102 (2018).

[40] V. Ivády, J. Davidsson, N. Delegan, A. L. Falk, P. V. Klimov, S. J. Whiteley, S. O. Hruszkewycz, M. V. Holt, F. J. Heremans, N. T. Son, *et al.*, Nat. Commun. **10**, 5607 (2019).

[41] T. Umeda, N. Son, J. Isoya, E. Janzén, T. Ohshima, N. Morishita, H. Itoh, A. Gali, and M. Bockstedte, Phys. Rev. Lett. **96**, 145501 (2006).

[42] M. Rosso, A. Arafat, K. Schroën, M. Giesbers, C. S. Roper, R. Maboudian, and H. Zuilhof, Langmuir **24**, 4007 (2008).

[43] Y. Li, S. Calder, O. Yaffe, D. Cahen, H. Haick, L. Kronik, and H. Zuilhof, Langmuir **28**, 9920 (2012).

[44] P. C. Lauterbur, M. H. M. Dias, and A. M. Rudin, in *Electrons to Tissues* (Elsevier, 1978) pp. 752–759.

[45] S. Steinert, F. Ziem, L. Hall, A. Zappe, M. Schweikert, N. Götz, A. Aird, G. Balasubramanian, L. Hollenberg, and J. Wrachtrup, Nat. Commun. **4**, 1607 (2013).

[46] T. Zhang, G. Pramanik, K. Zhang, M. Gulka, L. Wang, J. Jing, F. Xu, Z. Li, Q. Wei, P. Cigler, *et al.*, ACS Sens. **6**, 2077 (2021).

[47] T. Rendler, J. Neburkova, O. Zemek, J. Kotek, A. Zappe, Z. Chu, P. Cigler, and J. Wrachtrup, Nat. Commun. **8**, 14701 (2017).

[48] J. Wahsner, E. M. Gale, A. Rodríguez-Rodríguez, and P. Caravan, Chem. Rev. **119**, 957 (2018).

[49] M. Pelliccione, B. A. Myers, L. Pascal, A. Das, and A. B. Jayich, Phys. Rev. Appl. **2**, 054014 (2014).

[50] C. Li, M. Chen, D. Lyzwa, and P. Cappellaro, Nano Lett. **19**, 7342 (2019).

[51] J. Cai, A. Retzker, F. Jelezko, and M. B. Plenio, Nat. Phys. **9**, 168 (2013).

[52] G. Wolfowicz, F. J. Heremans, C. P. Anderson, S. Kanai, H. Seo, A. Gali, G. Galli, and D. D. Awschalom, Nat. Rev. Mater. **6**, 906 (2021).

[53] V. Ivády, K. Szász, A. L. Falk, P. V. Klimov, D. J. Christle, E. Janzén, I. A. Abrikosov, D. D. Awschalom, and A. Gali, Phys. Rev. B **92**, 115206 (2015).

[54] J. Cai, F. Jelezko, M. B. Plenio, and A. Retzker, New J. Phys. **15**, 013020 (2013).

[55] F.-F. Yan, J.-F. Wang, Q. Li, Z.-D. Cheng, J.-M. Cui, W.-Z. Liu, J.-S. Xu, C.-F. Li, and G.-C. Guo, Phys. Rev. Appl.

10, 044042 (2018).

[56] W.-X. Lin, F.-F. Yan, Q. Li, J.-f. Wang, Z.-H. Hao, J.-Y. Zhou, H. Li, L.-X. You, J.-S. Xu, C.-F. Li, *et al.*, Phys. Rev. B **104**, 125305 (2021).

[57] K. C. Miao, J. P. Blanton, C. P. Anderson, A. Bourassa, A. L. Crook, G. Wolfowicz, H. Abe, T. Ohshima, and D. D. Awschalom, Science **369**, 1493 (2020).
